# LINC01133 promotes hepatocellular carcinoma progression by sponging miR‐199a‐5p and activating annexin A2

**DOI:** 10.1002/ctm2.409

**Published:** 2021-05-06

**Authors:** Dan Yin, Zhi‐Qiang Hu, Chu‐Bin Luo, Xiao‐Yi Wang, Hao‐Yang Xin, Rong‐Qi Sun, Peng‐Cheng Wang, Jia Li, Jia Fan, Zheng‐Jun Zhou, Jian Zhou, Shao‐Lai Zhou

**Affiliations:** ^1^ Institute of Biomedical Sciences Fudan University Shanghai People's Republic of China; ^2^ Department of Liver Surgery and Transplantation, Liver Cancer Institute Zhongshan Hospital Fudan University Shanghai People's Republic of China; ^3^ Key Laboratory of Carcinogenesis and Cancer Invasion (Fudan University) Ministry of Education Shanghai People's Republic of China

**Keywords:** Annexin A2, EMT, hepatocellular carcinoma, LINC01133, miR‐199a‐5p

## Abstract

**Background:**

Long noncoding RNAs (lncRNAs) are functionally associated with cancer development and progression. Although gene copy number variation (CNV) is common in hepatocellular carcinoma (HCC), it is not known how CNV in lncRNAs affects HCC progression and recurrence. We aimed to identify a CNV‐related lncRNA involved in HCC progression and recurrence and illustrate its underlying mechanisms and prognostic value.

**Methods:**

We analyzed the whole genome sequencing (WGS) data of matched cancerous and noncancerous liver samples from 49 patients with HCC to identify lncRNAs with CNV. The results were validated in another cohort of 238 paired HCC and nontumor samples by TaqMan copy number assay. We preformed Kaplan‐Meier analysis and log‐rank test to identify lncRNA CNV with prognostic value. We conducted loss‐ and gain‐of‐function studies to explore the biological functions of LINC01133 in vitro and in vivo. The competing endogenous RNAs (ceRNAs) mechanism was clarified by microRNA sequencing (miR‐seq), quantitative real‐time PCR (qRT‐PCR), western blot, and dual‐luciferase reporter assays. We confirmed the binding mechanism between lncRNA and protein by RNA pull‐down, RNA immunoprecipitation, qRT‐PCR, and western blot analyses.

**Results:**

Genomic copy numbers of LINC01133 were increased in HCC, which were positively related with the elevated expression of LINC01133. Increased copy number of LINC01133 predicted the poor prognosis in HCC patients. LINC01133 overexpression in HCC cells promoted proliferation and aggressive phenotypes in vitro, and facilitated tumor growth and lung metastasis in vivo, whereas LINC01133 knockdown had the opposite effects. LINC01133 sponged miR‐199a‐5p, resulting in enhanced expression of SNAI1, which induced epithelial‐to‐mesenchymal transition (EMT) in HCC cells. In addition, LINC01133 interacted with Annexin A2 (ANXA2) to activate the ANXA2/STAT3 signaling pathway.

**Conclusions:**

LINC01133 promotes HCC progression by sponging miR‐199a‐5p and interacting with ANXA2. LINC01133 CNV gain is predictive of poor prognosis in patients with HCC.

## Graphical abstract

Genomic copy numbers of LINC01133 were increased in HCC, which were positively related with its elevated expression, and LINC01133 CNV gain predicted the poor prognosis in HCC patients.

LINC01133 sponged miR‐199a‐5p, resulting in enhanced expression of SNAI1, which induced EMT in HCC cells.

LINC01133 interacted with ANXA2 to activate ANXA2/STAT3 signaling pathway.

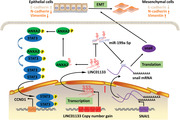

**Abbreviations**
ANXA2: Annexin A2
CCK‐8: cell‐counting‐8 kit
ceRNA: competing endogenous RNA
CNV: copy number variation
EMT: epithelial‐to‐mesenchymal transition
HCC: hepatocelluar carcinoma
ISH: in situ hybridization
lincRNA: long intergenic noncoding RNA
lncRNA: long noncoding RNA
miRNA: microRNA
OS: overall survival
qRT‐PCR: quantitative real‐time polymerase chain reaction
RIP: RNA immunoprecipitation
WGS: whole genome sequencing

## 1 BACKGROUND

The incidence and mortality rate of hepatocellular carcinoma (HCC) are rising worldwide and especially in China.^1^, ^2^ High rates of distant metastasis and recurrence lead to poor prognosis and a 5‐year survival rate below 50%.^3^ HCC development and progression involve complex genetic and epigenetic changes.^4^, ^5^, ^6^ Therefore, it is critical to understand those changes in order to identify prognostic predictors and therapeutic targets.

Copy number variation (CNV) is a type of structural variation involving deletions or amplifications of nucleotide sequences in specific regions of genomic DNA. CNV plays critical roles in cancer development and progression by activating oncogenes and inactivating tumor suppressors.^7^ Protein‐coding genes only account for 2% of the human genome, and numerous CNVs in noncoding genomic regions appear at a high frequency in populations.^8^ Long noncoding RNAs (lncRNAs) are transcribed RNA sequences at least 200 nucleotides in length that do not encode any protein.^9^ There is mounting evidence suggesting that lncRNAs function in many biological processes, including epigenetic, transcriptional, and posttranscriptional regulation.^10^, ^11^, ^12^ For example, lncRNAs exert regulatory functions in human disease development by binding to microRNAs (miRNAs) and suppressing miRNA‐mediated gene silencing.^13^ There is accumulating evidence suggesting that dysregulation of lncRNAs is involved in human cancers, including HCC.^14^, ^15^, ^16^ LncSox4 promotes early tumorigenesis in liver cells through the Stat3‐Sox4 pathway^11^; the lncRNA HULC promotes liver‐cancer progression by inhibiting PTEN via miR‐15a^17^; and the lncRNA MCM3AP‐AS1 promotes HCC through the miR‐194‐5p/FOXA1 axis and is associated with poor clinical outcomes in patients with HCC.^18^ CNV in lncRNAs such as focally amplified lncRNA on chromosome 1 (FAL1) affects lncRNA expression and can predict tumor prognosis.^19^ CNV in the lncRNA PRAL stimulates HCC growth, suggesting that PRAL might be an effective target for anticancer drug development.^20^ Long intergenic noncoding RNAs (lincRNAs) have no overlap with any other genes and are the most common type of lncRNAs.^21^ Therefore, it is of vital importance to explore the role of transcriptional dysregulation caused by CNV in lincRNAs during HCC progression and recurrence.

We analyzed the whole‐genome sequencing (WGS) data obtained in our previous study to screen for lincRNAs with CNV involved in HCC recurrence after curative resection.^22^ We examined CNVs and expression levels of candidate lincRNAs in HCC tissue samples. We then investigated the relationship between CNVs in lincRNAs and HCC outcomes. Finally, we explored the biological roles of the LINC01133 and the mechanisms by which it promotes HCC progression.

## 2 RESULTS

### 2.1 Identification of LINC01133 with CNV involved in HCC recurrence and prognosis

**FIGURE 1:**
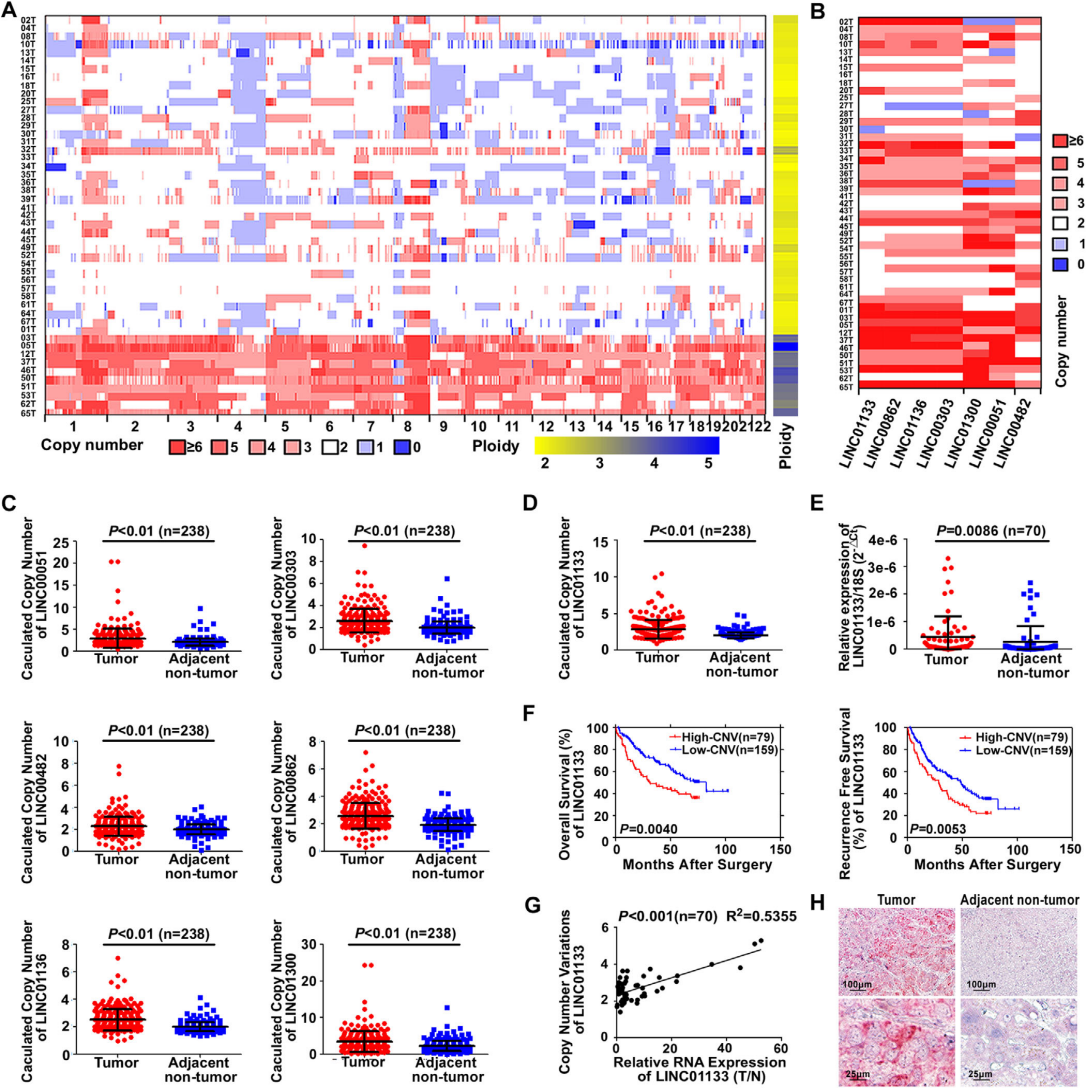
Identification of LINC01133 with CNV involved in HCC recurrence and prognosis. **(A)** A heatmap of genomic CNV in lncRNAs (cohort 1: *n* = 49). Chromosomal coordinates are on the *X*‐axis. The right band shows the ploidy status. **(B)** A heatmap of genomic CNV in seven selected lincRNAs (LINC00051, LINC00303, LINC00482, LINC00862, LINC01133, LINC01136, and LINC01300). **(C)** TaqMan copy number assay revealed that copy numbers of LINC00051, LINC00303, LINC00482, LINC00862, LINC01136, and LINC01300 were significantly increased in tumor tissues compared with those in adjacent nontumor tissues (cohort 2: *n* = 238). RNase P was used as an internal reference. **(D)** TaqMan copy number assay revealed that the copy number of LINC01133 was significantly increased in tumor tissues compared with that in adjacent nontumor tissues (cohort 2: *n* = 238). RNase P was used as an internal reference. **(E)** qRT‐PCR revealed that LINC01133 expression was increased in tumor tissues compared with that in adjacent nontumor tissues (*n* = 70). 18S rRNA was used as an internal reference. **(F)** Kaplan–Meier analysis of OS (left panel) and recurrence‐free survival (right panel) of 238 patients based on CNV in LINC01133 (cohort 2; the cutoff value for high‐and low‐​CNV was 3.0). **(G)** LINC01133 copy number and relative expression level were positively correlated in HCC tissues (*n* = 70), T: tumor tissue. N: adjacent nontumor tissue. **(H)** In situ hybridization results showed that LINC01133 was highly expressed in tumor tissues and mainly localized in the cytoplasm of HCC cells. Scale bar: 100 μm (upper panel), 25 μm (lower panel)

We revealed a genome‐wide heatmap of CNV in lncRNAs based on WGS data of 49 Chinese patients with HCC who experienced HCC recurrence after curative resection (cohort 1; Figures 1A and S1A).^22^ LncRNAs that were frequently amplified in HCC tumor tissues were mainly located on chromosomes 1q, 8q, 17q, and 20q, whereas lncRNAs that were frequently deleted in the tumor tissues were mainly located on chromosomes 4q, 9q, 13q, and 16q (Figure 1A). We selected seven lincRNAs (LINC00051, LINC00303, LINC00482, LINC00862, LINC01133, LINC01136, and LINC01300) that had average copy number ≥ 3.0 and CNV gain in > 50% of the HCC samples for further investigation (Figure 1B; Table S1). To verify the HCC‐related CNV of the selected lincRNAs, we analyzed matched tumors and nontumor tissues from additional 238 patients with HCC (cohort 2) by TaqMan copy number assay. The results confirmed that the copy numbers of all seven candidate lincRNAs were higher in the tumors than in the matched nontumor tissues (Figures 1C and D). We measured the seven lincRNAs expression in 70 pairs of tumor and nontumor samples randomly selected from cohort 2 and found that the expression levels of LINC00482 (*P *< 0.001), LINC00862 (*P *= 0.0014), and LINC01133 (*P *= 0.0086) were higher in the tumor samples than in the matched nontumor samples (Figures 1E and S1B). No significant differences in the expression levels of the other four candidate lincRNAs were found between the tumor and tumor‐adjacent tissues (Figure S1B).

Next, we analyzed the association between CNVs in the seven lincRNAs and clinical outcomes in cohort 2. We categorized the 238 patients into two groups, High‐CNV and Low‐CNV, according to each lincRNA CNVs (High‐CNV ≥ 3.0, Low‐CNV < 3.0; Figure S1C). Kaplan‐Meyer analysis showed that CNV gains in LINC01133 and LINC01300 were associated with reduced overall survival (OS; Figure 1F, left panel; Figure S1D). Because both the genomic copy numbers and the RNA expression of LINC01133 were increased in the tumors compared with those in the tumor‐adjacent tissues, we selected LINC01133 for further investigation. Patients with high LINC01133 copy numbers in their tumors exhibited shorter tumor‐free survival than those with low LINC01133 copy numbers in their tumors (Figure 1F, right panel). Univariate and multivariate analyses suggested that LINC01133 CNV was an independent prognostic factor for patient survival (Tables 1 and S2). In addition, the LINC01133 copy number had a positive relation with LINC01133 expression in the tumors relative to that in the tumor‐adjacent tissues (Figure 1G). RNAscope assay confirmed that LINC01133 was overexpressed in the HCC tumors compared with the tumor‐adjacent tissues and mainly existed in cytoplasm of the tumor cells (Figure 1H). Collectively, our results demonstrated that LINC01133 was frequently amplified at the genomic‐sequence level and overexpressed at the transcript level in the tumors of patients that experienced HCC, implying that LINC01133 plays a role in HCC recurrence and prognosis.

**TABLE 1:** Univariate and multivariate analyses of prognostic factors in HCC (Cohort 2, *n* = 238)

Variable	OS		TTR	
	HR (95% CI)	*P*	HR (95% CI)	*P*
**Univariate analysis** ^†^				
Age, year (≤50 vs > 50)	0.942(0.661‐1.341)	0.739	1.053(0.770‐1.439)	0.746
Sex (female vs male)	1.375(0.788‐2.399)	0.262	1.040(0.663‐1.631)	0.866
HBsAg (negative vs positive)	0.820(0.485‐1.387)	0.460	0.551(0.333‐0.912)	**0.020**
AFP, ng/mL (≤20 vs > 20)	1.284(0.880‐1.875)	0.195	1.145(0.826‐1.589)	0.416
GGT, U/L (≤54 vs > 54)	1.710(1.172‐2.494)	**0.005**	1.187(0.863‐1.631)	0.292
Liver cirrhosis (no vs yes)	1.405(0.893‐2.210)	0.142	1.349(0.907‐2.007)	0.139
Tumor size, cm (≤5 vs > 5)	2.927(2.003‐4.279)	**0.000**	2.090(1.518‐2.879)	**0.000**
Tumor number (single vs multiple)	1.178(0.742‐1.868)	0.487	1.206(0.801‐1.814)	0.369
Microvascular invasion (no vs yes)	2.713(1.639‐4.490)	**0.000**	2.000(1.235‐2.241)	**0.005**
Tumor encapsulation (complete vs none)	0.839(0.587‐1.199)	0.336	0.752(0.550‐1.029)	0.075
Tumor differentiation^‡^ (I + II vs III + IV)	1.620(1.130‐2.322)	**0.009**	1.201(0.868‐1.661)	0.269
CNV in LINC01133 (low vs high)	0.592(0.412‐0.850)	**0.004**	0.636 (0.461‐0.877)	**0.006**
**Multivariate analysis** ^†^				
HBsAg (negative vs positive)	NA	NA	0.722(0.561‐0.931)	**0.012**
GGT, U/L (≤54 vs > 54)	1.344(0.911‐1.983)	0.136	NA	NA
Tumor size, cm (≤5 vs > 5)	3.022(2.030‐4.500)	**0.000**	2.096(1.519‐2.893)	**0.000**
Microvascular invasion (no vs yes)	2.513(1.482‐4.263)	**0.001**	2.088(1.274‐3.424)	**0.004**
Tumor differentiation^‡^ (I + II vs III + IV)	1.595(1.104‐2.306)	**0.013**	NA	NA
CNV in LINC01133 (low vs high)	0.681(0.471‐0.986)	**0.042**	0.698(0.504‐0.967)	**0.030**

^†^ Analyses were conducted using univariate analysis or multivariate Cox proportional hazards regression.

^‡^ Edmondson grade. *P* values less than 0.05 were considered statistically significant. Boldface type indicates significant values. Abbreviations: OS, overall survival; TTR, time to recurrence; AFP, alpha‐fetoprotein; HBsAg, hepatitis B surface antigen; GGT, gamma‐glutamyl transpeptidase; HR, hazard ratio; CI, confidential interval; CNV: copy number variation; NA: not applicable.

### 2.2 Characteristics and distribution of LINC01133 in HCC cell lines

**FIGURE 2:**
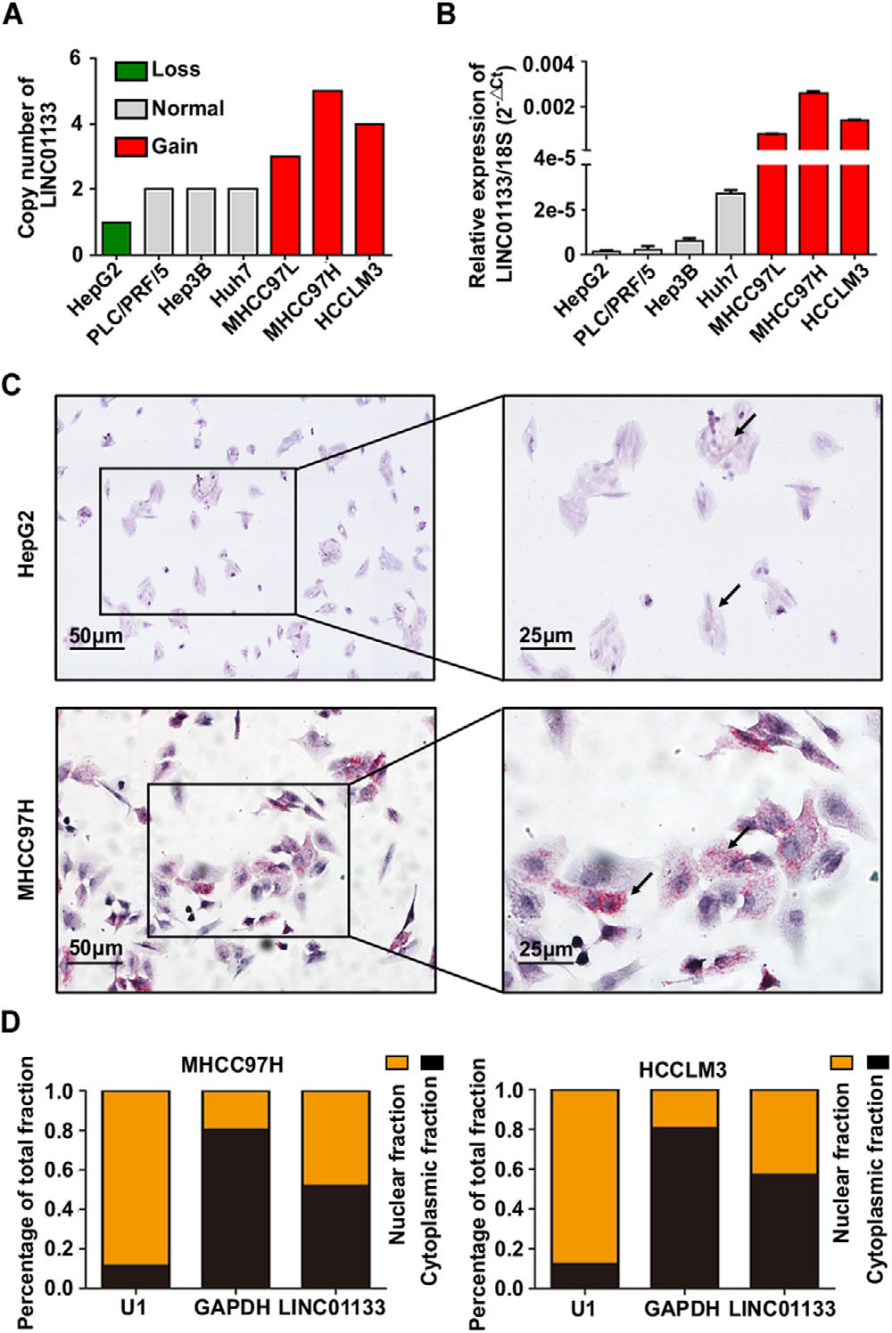
Characteristics and distribution of LINC01133 in HCC cell lines. **(A)** The copy numbers of LINC01133 in HCC cell lines determined by TaqMan copy number assay. RNase P was used as an internal reference. **(B)** Relative expression of LINC01133 in HCC cell lines determined by qRT‐PCR. 18S rRNA was used as an internal reference. Data are shown as mean ± SD and are representative of three independent experiments. **(C)** In situ hybridization experiments showed the localization and expression of LINC01133 in HepG2 (upper panel) and MHCC97H (lower panel) cells. The black arrows indicate the expression of LINC01133. Scale bar: 50 μm (left panel), 25 μm (right panel). **(D)** The subcellular localization of LINC01133 was detected by qRT‐PCR in MHCC97H and HCCLM3 cells. U1 snRNA was used as a nuclear reference. GAPDH was used as a cytoplasmic reference

LINC01133 is located on chromosome 1q23.2 (https://www.ncbi.nlm.nih.gov/gene/; Figure S2A) and has no protein‐encoding ability (https://lncipedia.org/; Table S3). We detected copy numbers and expression level of LINC01133 in HCC cell lines with different metastatic potentials and found that the LINC01133 copy numbers and expression level were greater in cell lines with high metastatic potential (MHCC97L, MHCC97H, and HCCLM3) than in cell lines with low metastatic potential (Hep3B, PLC/PRF/5, Huh7, and HepG2; Figures 2A and B). RNAscope assays showed that LINC01133 expression was higher in the MHCC97H cell line than in the HepG2 cell line and mainly existed in the cytoplasm in both cell lines (Figure 2C). Likewise, quantitative real‐time PCR (qRT‐PCR) revealed that although LINC01133 was present in both the cytoplasm and the nucleus of MHCC97H and HCCLM3 cells, it was relatively enriched in the cytoplasm of both cell lines (Figure 2D).

### 2.3 LINC01133 promotes aggressive HCC phenotypes in vitro and in vivo

**FIGURE 3:**
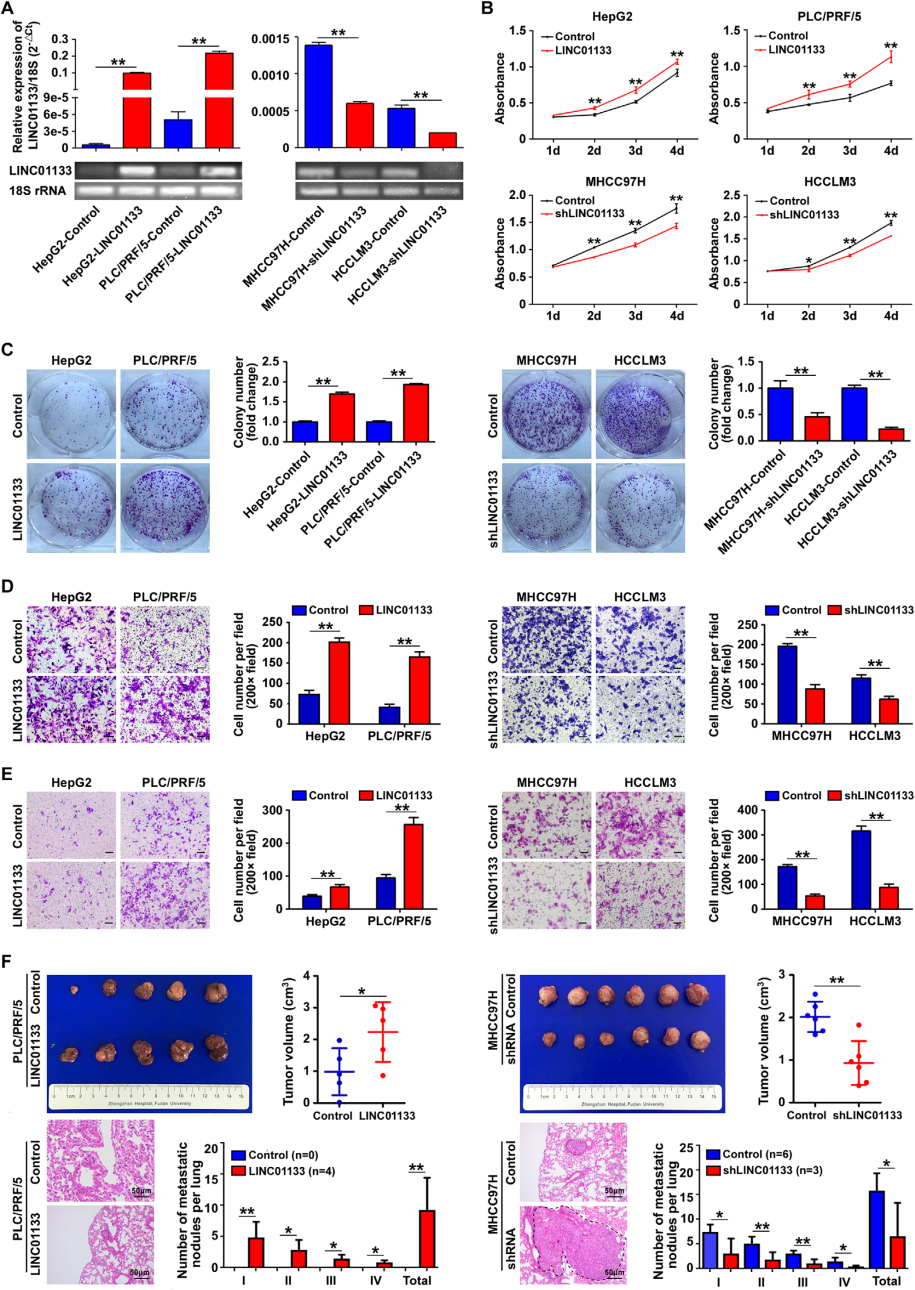
LINC01133 promotes HCC cell proliferation, migration, and invasion in vitro and in vivo. **(A)** qRT‐PCR and agarose gel electrophoresis assays showed that LINC01133 RNA levels increased in PLC/PRF/5 and HepG2 cells containing lentivirus‐based expression vectors (left panel) and decreased in MHCC97H and HCCLM3 cells transfected with an LINC01133‐specific shRNA (right panel). 18S rRNA was used as an internal reference, **P < 0.01. **(B)** CCK8 assays showed that LINC01133 overexpression promoted proliferation in HepG2 and PLC/PRF/5 cells (upper panel), whereas LINC01133 silencing inhibited proliferation in MHCC97H and HCCLM3 cells (down panel), *P < 0.05, **P < 0.01. **(C)** Colony‐formation assays showed that LINC01133 overexpression promoted colony formation in HepG2 and PLC/PRF/5 cells (left panel), whereas LINC01133 silencing inhibited colony formation in MHCC97H and HCCLM3 cells (right panel), **P < 0.01. **(D)** Cell‐migration assays showed that LINC01133 overexpression promoted migration in HepG2 and PLC/PRF/5 cells (left panel), whereas LINC01133 silencing inhibited migration in MHCC97H and HCCLM3 cells (right panel), **P < 0.01. Scale bar: 50 μm. **(E)** Cell‐invasion assays showed that LINC01133 overexpression promoted invasion in HepG2 and PLC/PRF/5 cells (left panel), whereas LINC01133 silencing inhibited invasion in MHCC97H and HCCLM3 cells (right panel), **P < 0.01. Scale bar: 50 μm. **(F)** Tumor weights of xenografts in the mouse model of HCC (upper panel). Representative images show hematoxylin‐eosin staining of lungs from different animal groups. The dotted circles indicate metastatic nodules in lung. The metastasis grades in each group are indicated (lower panel), *P < 0.05, **P < 0.01. Scale bar: 50 μm. All data are shown as the mean ± SD and are representative of three independent experiments

We used lentivirus‐based expression vectors to overexpress LINC01133 in the HepG2 and PLC/PRF/5 cell lines. In addition, we utilized short hairpin RNA (shRNA) to lower LINC01133 expression in the HCCLM3 and MHCC97H cell lines. We confirmed the overexpression (Figure 3A, left panel) and knockdown (Figure 3A, right panel) of LINC01133 in the cell lines by qRT‐PCR and agarose gel electrophoresis. Compared with those in corresponding control cells, proliferation and colony formation were both increased in the cells overexpressing LINC01133 and reduced in the cells with LINC01133 knockdown (Figures 3B and C). Similarly, the rates of migration and invasion were increased in the cells with LINC01133 overexpression and reduced in the cells with LINC01133 knockdown in comparison with those in corresponding control cells (Figures 3D and E).

Mouse xenograft experiments showed that PLC/PRF/5 cells overexpressing LINC01133 produced larger tumors and more metastatic lung nodules than PLC/PRF/5 cells expressing the empty vector. Conversely, MHCC97H cells with LINC01133 knockdown produced smaller tumors and fewer metastatic lung nodules than MHCC97H cells expressing a control shRNA (Figure 3F).

### 2.4 LINC01133 sponges miR‐199a‐5p in HCC cells

**FIGURE 4:**
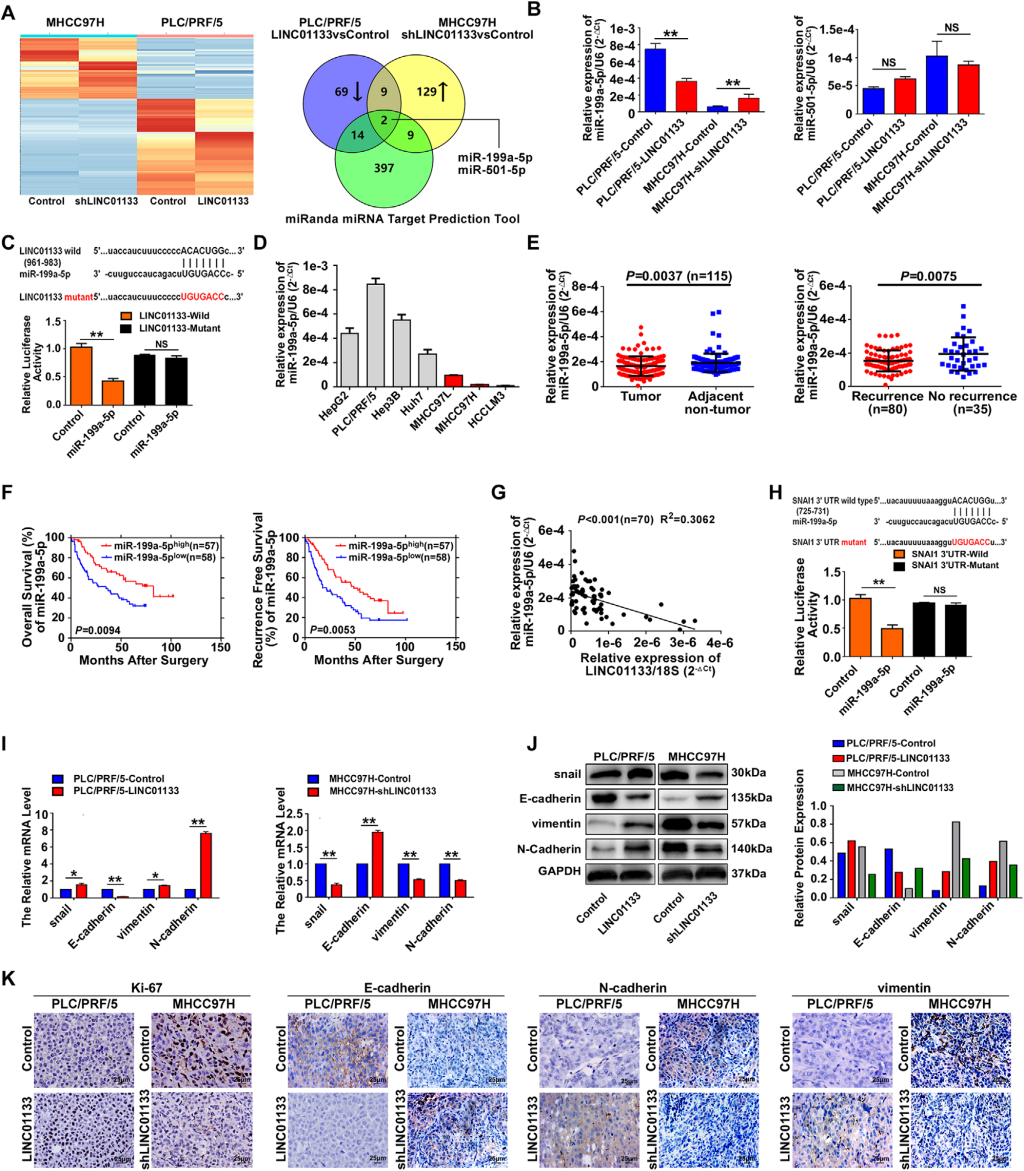
LINC01133 is a sponge of miR‐199a‐5p and induces EMT in HCC cells. **(A)** A heatmap (left panel) of differentially expressed miRNAs in LINC01133‐overexpressing PLC/PRF/5 cells versus control cells and LINC01133‐silenced MHCC97H cells versus control cells, as revealed by miR‐seq. Venn diagrams (right panel) show the numbers of miRNAs that potentially bind to LINC01133 according to three groupings: (1) downregulated miRNAs in PLC/PRF/5 cells after overexpression of LINC01133 (fold change > 2); (2) upregulated miRNAs in MHCC97H cells treated with shLINC01133 vectors (fold change > 2); and (3) miRNAs predicted by the miRNA Target Prediction Tool to interact with LINC01133. **(B)** qRT‐PCR was performed to examine the expression of miR‐199a‐5p (left panel) and miR‐501‐5p (right panel) in LINC01133‐overexpressing PLC/PRF/5 cells and LINC01133‐silenced MHCC97H cells. U6 snRNA was used as an internal reference, **P < 0.01. **(C)** The sequences of miR‐199a‐5p and its potential binding sites in LINC01133 are shown. The red nucleotides indicate mutant predicted binding sites. Luciferase assays were used to examine the interaction between LINC01133 and miR‐199a‐5p, **P < 0.01. **(D)** Relative expression of miR‐199a‐5p in HCC cell lines determined by qRT‐PCR. U6 snRNA was used as an internal reference. Data are shown as mean ± SD and are re*p*resentative of three independent experiments. **(E)** qRT‐PCR revealed that miR‐199a‐5p expression was decreased in tumor tissues compared with that in adjacent nontumor tissues (*n* = 115; left panel). qRT‐PCR revealed that miR‐199a‐5p expression was decreased in tumor tissues of HCC patients with recurrence (*n* = 80) compared with that in tumor tissues of HCC patients without recurrence (*n* = 35) (right panel). U6 snRNA was used as an internal reference. **(F)** Kaplan‐Meier analysis of OS (left panel) of 115 patients based on expression of miR‐199a‐5p (miR‐199a‐5p^high ^< median value, miR‐199a‐5p^low^ ≥ median value). Kaplan‐Meier analysis of recurrence‐free survival (right panel) of 115 patients based on expression of miR‐199a‐5p. **(G)** LINC01133 relative expression levels and miR‐199a‐5p relative expression levels were positively correlated in HCC tissues (*n* = 70). **(H)** The sequences of miR‐199a‐5p and its potential binding sites at the 3′UTR of SNAI1. The nucleotides mutated in the SNAI1‐3′UTR mutant are shown in red. Luciferase assays were conducted to examine the miR‐199a‐5p–SNAI1 interactions. qRT‐PCR **(I)** and western blot **(J)** showed changes in snail and EMT‐marker expression following stable upregulation or downregulation of LINC01133 expression. **(K)** Representative examples of Ki‐67, E‐cadherin, N‐cadherin, and vimentin immunohistochemistry from PLC/PRF/5‐Control, PLC/PRF/5‐LINC01133, MHCC97H‐Control, and MHCC97H‐shLINC01133‐derived xenografts tissues

LincRNAs in the cytoplasm can act as sponges to inactivate miRNAs and thus regulate the posttranscriptional translation of target genes.^13^ To determine if LINC01133 can sponge miRNAs to affect HCC development and progression, we performed miRNA sequencing (miR‐seq) in HCC cells with LINC01133 overexpression and LINC01133 silencing. Among 1620 known miRNAs appearing in one or more of the HCC cell lines, we identified 94 miRNAs that were downregulated (fold‐change > 2) in PLC/PRF/5 cells overexpressing LINC01133 in comparison with control cells, 149 miRNAs that were upregulated (fold‐change > 2) in MHCC97H cells with LINC01133 silencing in comparison with control cells, and 422 miRNAs that were predicted by the miRanda miRNA Target Prediction Tool to bind to LINC01133 (Figure 4A). Two miRNAs, miR‐199a‐5p and miR‐501‐5p, were included in all three groups (Figure 4A, right panel). qRT‐PCR showed that LINC01133 overexpression reduced miR‐199a‐5p levels rather than miR‐501‐5p levels, in PLC/PRF/5 cells (Figure 4B). Conversely, LINC01133 knockdown enhanced miR‐199a‐5p expression rather than miR‐501‐5p, in MHCC97H cells (Figure 4B). Analysis using the miRanda software revealed sequence complementary to miR‐199‐5p at nucleotide positions 961‐983 of LINC01133 (Figure 4C). In dual‐luciferase reporter assays, miR‐199a‐5p inhibited the luciferase activity of wild‐type LINC01133 but not that of mutant LINC01133 (Figure 4C). These data indicated that LINC01133 sponges miR‐199a‐5p in HCC cells.

### 2.5 The expression and prognostic value of miR‐199a‐5p in HCC

We detected the miR‐199a‐5p expression in HCC cell lines and found that miR‐199a‐5p expression was lower in HCC cell lines with high metastatic potential (MHCC97L, MHCC97H, and HCCLM3) than in cell lines with low metastatic potential (Hep3B, PLC/PRF/5, Huh7, and HepG2; Figure 4D). We also measured miR‐199a‐5p expression in 115 pairs of tumor and adjacent nontumor samples randomly selected from cohort 2. qRT‐PCR showed that miR‐199a‐5p expression was lower in the tumors than in the matched peritumoral samples (*P *= 0.0037; Figure 4E, left panel), and also lower in the tumor tissues of patients with HCC recurrence than those without recurrence (*P *= 0.0075; Figure 4E, right panel).

Then we explored the prognostic value of miR‐199a‐5p expression in HCC. Kaplan‐Meyer analysis showed that high miR‐199a‐5p levels were associated with increased OS (miR‐199a‐5p^high ^> median value, miR‐199a‐5p^low^ ≤ median value; Figure 4F, left panel). Patients with high miR‐199a‐5p expression in their tumors exhibited longer tumor‐free survival than those with low miR‐199a‐5p in their tumor (Figure 4F, right panel). In addition, we found that the miR‐199a‐5p expression level had a negative relation with the LINC01133 expression level in tumors (Figure 4G). These results suggested that LINC01133 facilitated HCC development and progression by suppressing the tumor‐suppressor activity of miR‐199a‐5p via a lincRNA‐miRNA sponging mechanism.

### 2.6 LINC01133 triggers epithelial‐to‐mesenchymal transition (EMT) in HCC through miR‐199a‐5p/snail signaling

It has been reported that miR‐199a‐5p inhibits SNAI1 expression by binding to the 3′‐untranslated region (UTR) of SNAI1 transcripts.^23^, ^24^ In agreement with a previous study, analysis using TargetScan 7.0 (http://targetscan.org) indicated that the 3′‐UTR of the SNAI1 mRNA contains a sequence that is complementary to miR‐199a‐5p (Figure 4H). Luciferase reporter assays showed that miR‐199a‐5p overexpression reduced the reporter activity of the wild‐type SNAI1 3′‐UTR, but not that of a mutant SNAI1 3′‐UTR (Figure 4H). It is well known that snail is a key transcription factor linked to EMT. Therefore, we examined the expression of snail and markers related to EMT in HCC cells with LINC01133 overexpression or knockdown by qRT‐PCR and western blot. We found that LINC01133 overexpression in PLC/PRF/5 cells enhanced the expression of snail, vimentin, and N‐cadherin at both the mRNA level and the protein level, whereas LINC01133 knockdown in MHCC97H cells reduced the mRNA and proteins levels of those EMT markers (Figures 4I and J). LINC01133 expression had the inverse effects on E‐cadherin expression (Figures 4I and J). In vivo, we found that LINC01133 overexpression in PLC/PRF/5 cell‐derived xenografts upregulated the protein levels of Ki‐67, vimentin, and N‐cadherin, whereas LINC01133 knockdown in MHCC97H cell‐derived xenografts attenuated the proteins levels of Ki‐67, vimentin, and N‐cadherin (Figure 4K). Similar to the in vitro results, LINC01133 expression in vivo had the inverse effects on E‐cadherin expression (Figure 4K).

**FIGURE 5:**
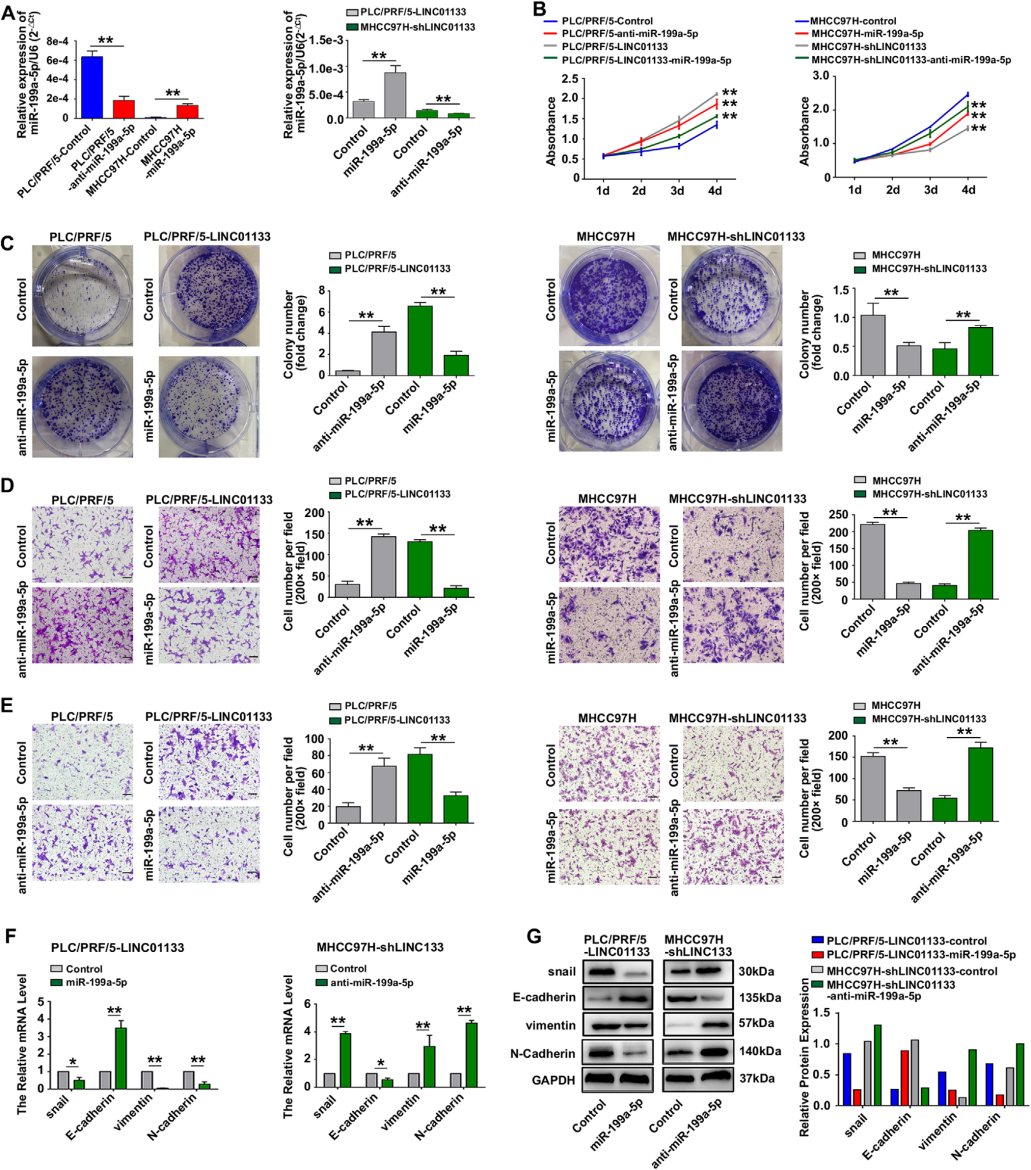
LINC01133 plays functions via miR‐199a‐5p‐dependent manner. **(A)** qRT‐PCR was performed to assess the expression of miR‐199a‐5p in PLC/PRF/5 cells and MHCC97H cells following treatment with anti‐miR‐199a‐5p and miR‐199a‐5p, respectively (left panel). qRT‐PCR was performed to assess the expression of miR‐199a‐5p in LINC01133‐overexpressing PLC/PRF/5 cells and LINC01133‐silenced MHCC97H cells following treatment with miR‐199a‐5p and anti‐miR‐199a‐5p, respectively (right panel). U6 snRNA was used as an internal reference. **P < 0.01. **(B)** CCK8 assays showed that LINC01133 overexpression and miR‐199a‐5p silencing promoted proliferation in PLC/PRF/5 cells, and miR‐199a‐5p overexpression inhibited proliferation in LINC01133‐overexpressing PLC/PRF/5 cells (left panel). LINC01133 silencing and miR‐199a‐5p overexpression inhibited proliferation in MHCC97H cells, and miR‐199a‐5p silencing promoted proliferation in LINC01133‐silenced MHCC97H cells (right panel). *P < 0.05, **P < 0.01. **(C)** Colony‐formation assays showed that LINC01133 overexpression and miR‐199a‐5p silencing promoted colony formation in PLC/PRF/5 cells, and miR‐199a‐5p overexpression inhibited colony formation in LINC01133‐overexpressing PLC/PRF/5 cells (left panel). LINC01133 silencing and miR‐199a‐5p overexpression inhibited colony formation in MHCC97H cells, and miR‐199a‐5p silencing promoted colony formation in LINC01133‐silenced MHCC97H cells (right panel). **P < 0.01. **(D)** Cell‐migration assays showed that LINC01133 overexpression and miR‐199a‐5p silencing promoted migration in PLC/PRF/5 cells, and miR‐199a‐5p overexpression inhibited migration in LINC01133‐overexpressing PLC/PRF/5 cells (left panel). LINC01133 silencing and miR‐199a‐5p overexpression inhibited migration in MHCC97H cells, and miR‐199a‐5p silencing promoted migration in LINC01133‐silenced MHCC97H cells (right panel). **P < 0.01. Scale bar: 50 μm. **(E)** Cell‐invasion assays showed that LINC01133 overexpression and miR‐199a‐5p silencing promoted invasion in PLC/PRF/5 cells, and miR‐199a‐5p overexpression inhibited invasion in LINC01133‐overexpressing PLC/PRF/5 cells (left panel). LINC01133 silencing and miR‐199a‐5p overexpression inhibited invasion in MHCC97H cells, and miR‐199a‐5p silencing promoted invasion in LINC01133‐silenced MHCC97H cells (right panel). **P < 0.01. Scale bar: 50 μm. qRT‐PCR **(F)** and western blot **(G)** were conducted to measure mRNA and protein levels of snail, E‐cadherin, vimentin, and N‐cadherin in LINC01133‐overexpressing PLC/PRF/5 cells and LINC01133‐silenced MHCC97H cells after treatment with miR‐199a‐5p and anti‐miR‐199a‐5p, respectively. GAPDH was used as an internal reference in qRT‐PCR and western blot. Data are shown as the mean ± SD and are representative of three independent experiments

For further investigation, we performed rescue experiments to determine whether LINC01133 plays a biological role and regulates EMT in an miR‐199a‐5p‐dependent manner. We knocked down miR‐199a‐5p expression in wild‐type PLC/PRF/5 cells and LINC01133‐silenced MHCC97H cells by transfecting them with an anti‐miR‐199a‐5p lentiviral vector. In addition, we overexpressed miR‐199a‐5p in wild‐type MHCC97H cells and LINC01133‐overexpressing PLC/PRF/5 cells by transfecting them with an miR‐199a‐5p lentiviral vector. We validated the expression of miR‐199a‐5p in each cell line by qRT‐PCR (Figure 5A). The miR‐199a‐5p knockdown enhanced proliferation and colony formation in PLC/PRF/5 cells and LINC01133‐silenced MHCC97H cells in comparison with control cells without miR‐199a‐5p knockdown. Conversely, miR‐199a‐5p overexpression suppressed proliferation and colony formation in MHCC97H cells and LINC01133‐overexpressing PLC/PRF/5 cells in comparison with control cells without miR‐199a‐5p overexpression (Figures 5B and C). Similarly, miR‐199a‐5p knockdown increased migration and invasion in PLC/PRF/5 cells and LINC01133‐silenced MHCC97H cells, whereas miR‐199a‐5p overexpression reduced migration and invasion in MHCC97H cells and LINC01133‐overexpressing PLC/PRF/5 cells (Figures 5D and E).

Furthermore, miR‐199a‐5p overexpression in LINC01133‐overexpressing PLC/PRF/5 cells resulted in the upregulation of E‐cadherin and the downregulation of snail, vimentin, and N‐cadherin, whereas miR‐199a‐5p knockdown had the inverse effects on the expression of those proteins in LINC01133‐silenced MHCC97H cells (Figures 5F and G). Taken together, these results suggested that LINC01133 plays biological functions and regulates EMT in HCC cells in an miR‐199a‐5p‐dependent manner.

### 2.7 LINC01133 binds to ANXA2 in HCC cells

**FIGURE 6:**
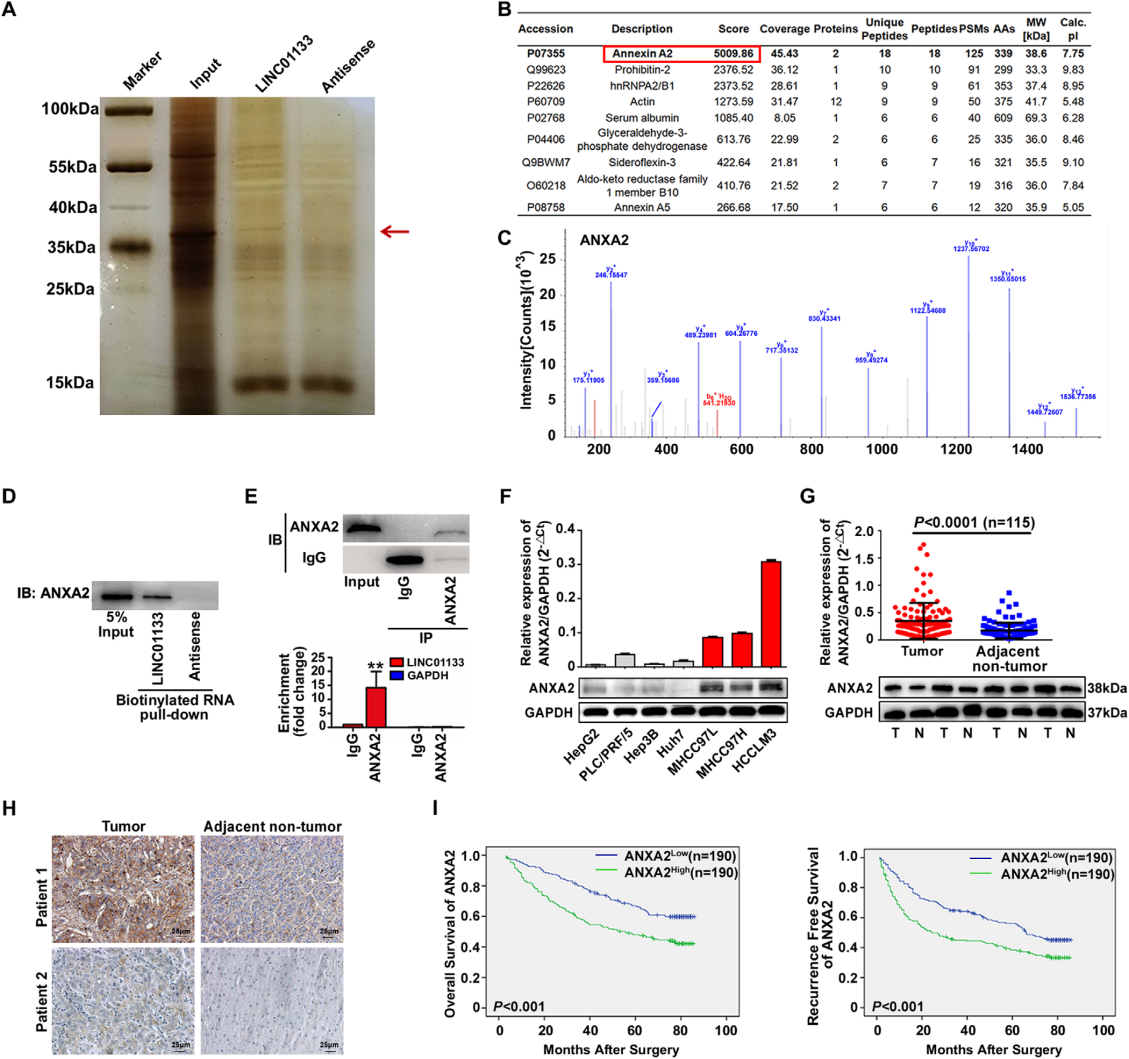
LINC01133 interacts with ANXA2 in HCC cells. **(A)** RNA pull‐down assay was conducted to identify proteins that physically interact with LINC01133. After silver staining, the bands around 35 kDa (red arrow) were cut and analyzed by mass spectrometry. **(B)** The top nine selected proteins that potentially interact with LINC01133 are shown. **(C)** The secondary mass spectrum of ANXA2 identified by mass spectrometry. **(D)** Western blot analysis of LINC01133‐bound ANXA2 from the RNA pull‐down assay (bottom). **(E)** RNA immunoprecipitation (RIP) was performed to verify LINC01133–ANXA2 interaction. qRT‐PCR was conducted to detect LINC01133 enrichment. IgG protein and GAPDH were used as negative controls for RIP and qRT‐PCR, respectively. **P < 0.01. **(F)** Relative expression of ANXA2 in HCC cell lines determined by qRT‐PCR and western blot. GAPDH was used as an internal reference. Data are shown as the mean ± SD and are representative of three independent experiments. **(G)** qRT‐PCR revealed that ANXA2 mRNA expression was increased in tumor tissues compared with that in adjacent nontumor tissues (*n* = 115). Western blot revealed that ANXA2 protein expression was increased in tumor tissues compared with that in adjacent nontumor tissues. GAPDH was used as an internal reference. T: tumor tissue. N: adjacent nontumor tissue.**(H)** Representative examples of immunohistochemistry showed that the expression of ANXA2 was high in HCC tumor tissues compared with that in adjacent nontumor tissues. **(I)** Kaplan‐Meier analysis of overall survival (left panel) and recurrence‐free survival (right panel) of 380 patients based on expression of ANXA2 (ANXA2^high^ ≥ median value, ANXA2^low^ < median value)

In addition to interacting with other RNAs, lincRNAs exert functions by interacting directly with proteins. We performed RNA pull‐down assays to identify proteins that can bind directly to LINC01133. Mass spectrometry analysis of a ∼35 KD protein band pulled down by LINC01133 (Figure 6A) revealed nine potential LINC01133‐binding proteins, excluding keratin, with peptide number > 5 and unique peptide number > 5 (Figure 6B). Of the nine potential LINC01133‐binding proteins, ANXA2 had the highest total score in a protein‐identification analysis (Figures 6B and C). We verified the presence of ANXA2 among the proteins isolated in the RNA pull‐down assay by western blot (Figure 6D). The RNAfold database (http://rna.tbi.univie.ac.at/cgibin/RNAWebSuite/RNAfold.cgi) and Cat‐Rapid database (http://service.tartaglialab.com/page/catrapid_omics_group) consistently predicted that ANXA2 binds to nucleotides 126‐177 or nucleotides 651‐792 of LINC01133 (Figures S2B and S2C). Moreover, RNA immunoprecipitation (RIP) assays showed that LINC01133 was enriched in lysates of MHCC97H cells treated with anti‐ANXA2 antibody in comparison with lysates of MHCC97H cells treated with IgG protein (Figure 6E), further confirming the interaction between LINC01133 and ANXA2 in HCC cells.

### 2.8 LINC01133 activates ANXA2/STAT3/cyclin D1 signaling in HCC cells

We measured ANXA2 expression in HCC cell lines and found that ANXA2 expression was higher in HCC cell lines with high metastatic potential (MHCC97L, MHCC97H, and HCCLM3) than in cell lines with low metastatic potential (Hep3B, PLC/PRF/5, Huh7, and HepG2; Figure 6F). Then, we measured ANXA2 expression in 115 pairs of tumors and adjacent nontumor samples randomly selected from cohort 2. qRT‐PCR showed that ANXA2 mRNA expression was higher in the HCC tumors than in the matched peritumoral tissues (*P *< 0.0001; Figure 6G). Western blot and immunohistochemistry analyses confirmed that the ANXA2 protein levels were increased in HCC tumors compared with those in matched nontumor tissues (Figures 6G and H).

To explore the prognostic value of ANXA2 in HCC, we detected ANXA2 expression in Tissue Microarray Technique (TMA; cohort 3, *n* = 380). We divided cohort 3 into two groups based on the median ANXA2 expression (ANXA2^high ^≥ ​median value, ANXA2^low^ < median value). Kaplan‐Meyer analysis showed that high ANXA2 expression was associated with reduced OS (Figure 6I, left panel). Patients with high ANXA2 expression in their tumors exhibited shorter tumor‐free survival than those with low ANXA2 expression in their tumors (Figure 6I, right panel).

**FIGURE 7:**
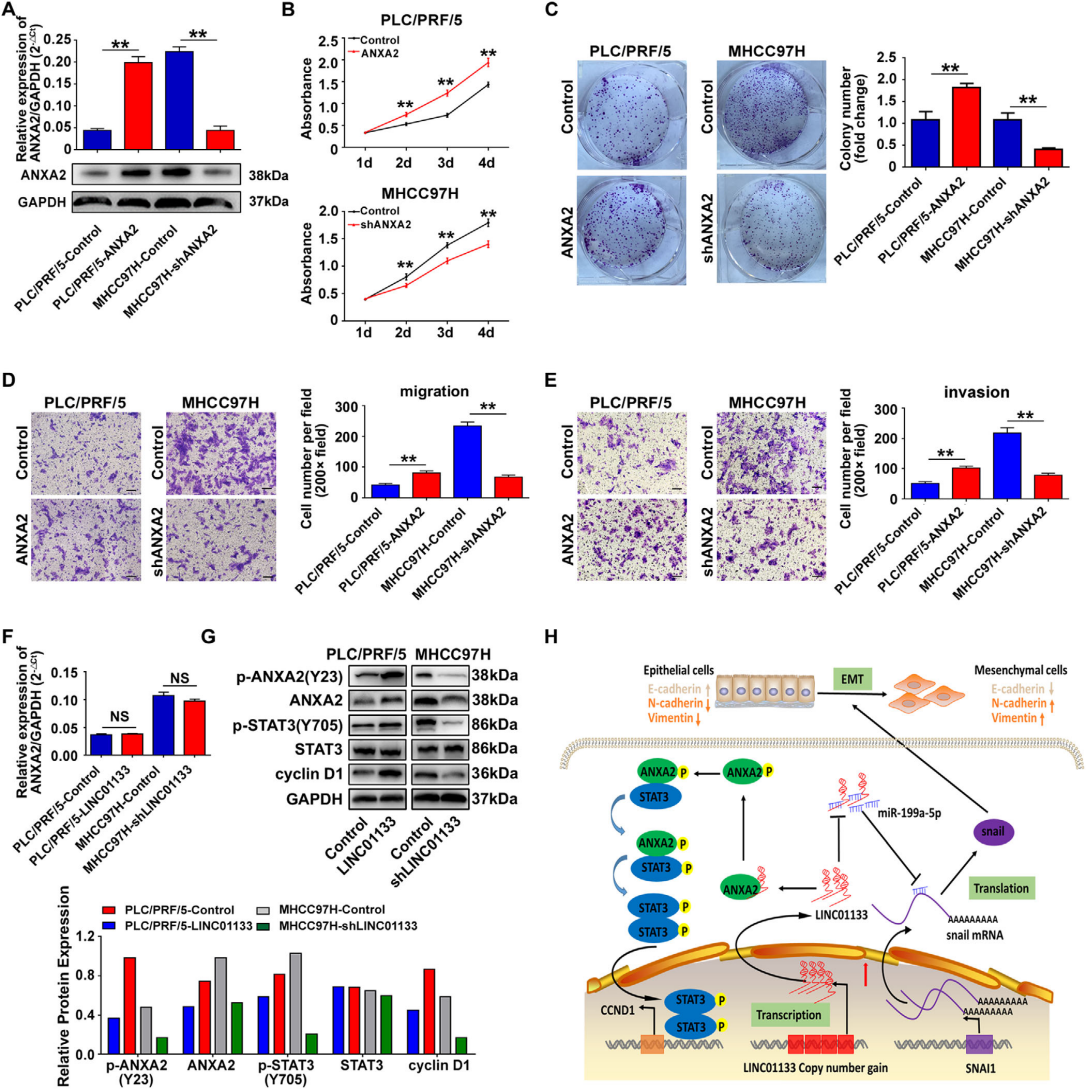
ANXA2 promotes HCC cell proliferation, migration, and invasion in vitro. **(A)** qRT‐PCR and western blot were conducted to measure expression of ANXA2 in ANXA2‐overexpressing PLC/PRF/5 cells and ANXA2‐silenced MHCC97H cells. GAPDH was used as an internal reference. **(B)** CCK8 assays showed that ANXA2 overexpression promoted proliferation in PLC/PRF/5 cells (upper panel), whereas ANXA2 silencing inhibited proliferation in MHCC97H cells (down panel). **P < 0.01. **(C)** Colony‐formation results showed that ANXA2 overexpression promoted colony formation in PLC/PRF/5 cells (left panel), whereas ANXA2 silencing inhibited colony formation in MHCC97H cells (right panel). **P < 0.01. **(D)** Cell‐migration assays showed that ANXA2 overexpression promoted migration in PLC/PRF/5 cells (left panel), whereas ANXA2 silencing inhibited migration in MHCC97H cells (right panel). **P < 0.01. Scale bar: 50 μm. **(E)** Cell‐invasion assays showed that ANXA2 overexpression promoted invasion in PLC/PRF/5 cells (left panel), whereas ANXA2 silencing inhibited invasion in MHCC97H cells (right panel). **P < 0.01. Scale bar: 50 μm. **(F)** qRT‐PCR was conducted to measure mRNA levels of ANXA2 in LINC01133‐overexpressing PLC/PRF/5 cells and LINC01133‐silenced MHCC97H cells. GAPDH was used as an internal reference. **(G)** Western blot analysis was used to assess protein expression of ANXA2, p‐ANXA2, STAT3, p‐STAT3, and cyclinD1 following stable upregulation or downregulation of LINC01133. GAPDH was used as an internal control. Data are shown as the mean ± SD and are representative of three independent experiments. **(H)** Schematic diagram of a proposed model of the regulation of LINC01133 in HCC

Next, we overexpressed ANXA2 in PLC/PRF/5 cells and knocked down ANXA2 expression in MHCC97H cells (Figure 7A). Compared with those in corresponding control cells, proliferation and colony formation were increased in the PLC/PRF/5 cells overexpressing ANXA2 and decreased in the MHCC97H cells with ANXA2 knockdown (Figures 7B and C). Similarly, rate of migration and invasion was increased in cells overexpressing ANXA2 and reduced in cells with ANXA2 knockdown (Figures 7D and E).

To further investigate the possible role of the LINC01133/ANXA2 signaling axis in HCC, we measured the expression of ANXA2 by qRT‐PCR and western blot. The mRNA level of ANXA2 was not affected by LINC01133 overexpression in PLC/PRF/5 cells or by LINC01133 silencing in MHCC97H cells (Figure 7F). By contrast, the protein level of ANXA2 was increased by LINC01133 overexpression in PLC/PRF/5 cells and reduced by LINC01133 silencing in MHCC97H cells (Figure 7G). Furthermore, LINC01133 overexpression increased the phosphorylation levels of ANXA2 and STAT3 and the expression level of cyclin D1 in PLC/PRF/5 cells, whereas LINC01133 silencing reduced the phosphorylation levels of ANXA2 and STAT3 and the expression level of cyclin D1 in MHCC97H cells (Figure 7G). Taken together, our results suggested that LINC01133 interacts with ANXA2 to activate STAT3/cyclin D1 signaling, which may be involved in the progression of HCC induced by this lincRNA.

## 3 DISCUSSION

Genomic and molecular‐phenotypic heterogeneity due to CNV is thought to influence HCC development and progression.^7^ Many examples of CNV have been identified in intergenic regions of DNA in human HCC cells.^19^, ^20^, ^25^ In this study, based on WGS and bioinformatics analyses of 49 matched HCC samples and para‐cancer tissues, the genomic CNV of lncRNAs in Chinese patients with early HCC recurrence after curative resection was revealed for the first time. In a previous study, Yang et al identified frequent deletions in 147 lncRNAs, such as FENDRR, that are recurrently deregulated in HCC.^26^ Li et al identified frequent sequence amplifications in the oncogenic lncRNA LINC01138 in HCC and showed that the amplifications were associated with poor disease outcomes.^27^ Zhou et al reported that CNV in the lncRNA PRAL stimulated HCC proliferation and might provide an effective target for antitumor therapy.^20^ We found that LINC01133 copy numbers and expression were elevated in HCC compared with those in matched nontumor tissues. Furthermore, LINC01133 expression was positively correlated with the LINC01133 copy number in HCC samples, suggesting that LINC01133 increased copy number is at least partially responsible for the frequent LINC01133 upregulation in HCC. A previous study reported that hypomethylation of lncRNA genes may have an effect on increased expression of lncRNAs such as SOX21‐AS1 in cervical cancer.^28^ Hence, the increased LINC01133 expression observed in our study might be caused by DNA hypomethylation in addition to copy number gains. Furthermore, LINC01133 CNV gain was predictive of early HCC recurrence and poor prognosis after curative surgery. These findings suggest that CNV in LINC01133 plays a role in HCC recurrence and prognosis.

There is accumulating evidence that the downstream targets and signaling pathways regulated by LINC01133 contributes to the malignant behavior of multiple types of cancer cells.^29^, ^30^, ^31^, ^32^ In nonsmall cell lung carcinoma, LINC01133 suppresses the transcription of KLF2, P21, and E‐cadherin by binding to EZH2 and LSD1, resulting in increased cancer cell growth and invasion.^33^ In colon cancer, LINC01133 inhibits EMT and metastasis by interacting with SRSF6.^34^ In osteosarcoma, LINC01133 promotes tumor growth as a miR‐422a sponge.^31^ In our study, we revealed that LINC01133 promotes aggressive phenotypes in HCC cell lines and in mouse xenograft models. Furthermore, we explored the underlying mechanism of LINC01133 in HCC progression.

LincRNAs interact with proteins, miRNAs, and other molecules to exert their functions.^35^ Previous studies suggested that LINC01133 sponges miR‐4784 and miR‐205 to counter miRNA‐mediated gene silencing in cervical cancer and gastric cancer, respectively.^36^, ^37^ Our miR‐seq and bioinformatics analyses revealed that LINC01133 sponges miR‐199a‐5p in HCC. We also predicted on the basis of bioinformatic analysis that LINC01133 interacts with miR‐501‐5p, but qRT‐PCR revealed that LINC01133 did not affect the expression of miR‐501‐5p in HCC cells. A previous study suggested that the sponging of miRNAs has a reciprocal effect on lncRNA expression; miR‐34a directly binds and thus reduces the half‐life of lncRNA‐UFC1, which prior to binding to miR‐34a has a stimulating effect on HCC cell proliferation.^38^ MiR‐501‐5p might similarly influence the expression of LINC01133.

Additionally, our results confirmed previous findings that SNAI1 is a target of miR‐199a‐5p.^23^ Rescue assays showed that LINC01133 upregulates snail expression by sponging miR‐199a‐5p. EMT is a key factor in tumor invasion and metastasis.^39^ Features of EMT are the gain of mesenchymal markers, such as vimentin and N‐cadherin, and the loss of epithelial cell‐junction proteins, such as E‐cadherin, in subsets of cancer cells.^40^ We reported that high LINC01133 expression in HCC cells was correlated with high vimentin and N‐cadherin expression and low E‐cadherin expression and, furthermore, that LINC01133 induced EMT in HCC cells by sponging miR‐199a‐5p.

LincRNAs can interact with proteins to regulate gene expression at the posttranslational level. LINC01133 exerts oncogenic functions in nasopharyngeal carcinoma and lung cancer by binding to YBX1 and EZH2, respectively.^33^, ^41^ We revealed that LINC01133 bound to ANXA2 to promote the expression of that protein. Several studies demonstrated that Tyr23 phosphorylation of ANXA2 enhances cancer proliferation and metastasis.^42^, ^43^ ANXA2 also regulates glioma cell proliferation via the STAT3‐cyclin D1 pathway.^44^ We found that LINC01133 increased the total protein level of ANXA2 and the phosphorylation levels of ANXA2 and STAT3 and also upregulated the downstream target gene cyclin D1 in HCC, which might account for its effects on HCC progression.

## 4 CONCLUSION

LINC01133 CNV gain is predictive of poor prognosis in HCC. LINC01133 promotes HCC progression by sponging miR‐199a‐5p to enhance snail expression, resulting in enhanced EMT. LINC01133 can also interact with ANXA2 to activate STAT3/cyclin D1 signaling (Figure 7H). Therefore, LINC01133 might be a new indicator for HCC progression and prognosis and a potential therapeutic target in HCC.

## 5 MATERIALS AND METHODS

### 5.1 Patients and follow up

In total, 667 patients with primary HCC were included in three independent cohorts (cohort 1, *n* = 49; cohort 2, *n* = 238; cohort 3, *n* = 380). All the patients underwent curative resection at Zhongshan Hospital affiliated with Fudan University (Shanghai, China). The patient characteristics are summarized in Table S4. Patients who had palliative surgery only, prior interventions for HCC, primary malignancies other than HCC, or inflammatory diseases were excluded from cohort 1. And all patients in cohort 1 experienced HCC early recurrence after curative surgery. In cohort 1, snap‐frozen samples of primary tumors and tumor‐adjacent liver tissues were obtained during curative resection between 2010 and 2011 and used for WGS. In cohort 2, snap‐frozen tissue specimens from another 238 consecutive patients with primary HCC were surgically removed in 2010, and used for TaqMan copy number assay and qRT‐PCR analysis. In cohort 3, tissue samples, embedded in paraffin, were obtained from 380 patients who were randomly selected and underwent curative resection for HCC in 2006.^45^ Postoperative follow‐up of cohort 1 and cohort 2 was conducted until June 30, 2016. Postoperative follow‐up of cohort 3 was conducted until March 15, 2013.

All patients included in the study were histopathologically diagnosed with HCC according to World Health Organization criteria. The Edmondson and Steiner classification system was used to determine the tumor grade.^46^ Liver function in each participant at the time of surgery was classified according to the Child‐Pugh system. The Research Ethics Committee of Zhongshan Hospital reviewed and approved the use of human subjects, and each participant provided informed consent for inclusion in the study. Tumor recurrence was determined by computed tomography, magnetic resonance imaging, digital subtraction angiography, and serum alpha‐fetoprotein level, with or without histological confirmation.^47^ We defined early recurrence as recurrence within the first 2 years after surgery.^48^ The definition of time to recurrence was the time period between surgery and diagnosis of intrahepatic recurrence or distant metastasis.^49^ We defined OS as the time period between surgery and death due to any cause or the end of follow‐up. We censored the data of surviving patients at the last follow‐up.

### 5.2 Cell lines and nude mice

The isogenic human HCC cell lines MHCC97L, MHCC97H, and HCCLM3 were established in our institute and display stepwise increases in their potential to metastasize to the lungs from xenografts in mice.^50^ The human HCC cell lines Hep3B, HepG2, PLC/PRF/5, and Huh7 have low metastatic potential and were obtained from the Shanghai Institute of Biochemistry and Cell Biology, Chinese Academy of Sciences (Shanghai, China). All cell lines were grown in Dulbecco's modified Eagle's medium (DMEM; Gibco, CA, USA) with 10% fetal bovine serum (FBS; Gibco) at 37°C in an incubator with 5% CO_2_.

Male BALB/c nu/nu (4‐6 weeks old) mice were obtained from the Shanghai Institute of Material Medicine, Chinese Academy of Science (Shanghai, China). We reared the nude mice under specific pathogen‐free conditions. We followed the “Guide for the Care and Use of Laboratory Animals” to treat nude mice humanely, and the guide was published by the National Institutes of Health (NIH publication 86‐23 revised 1985).

### 5.3 WGS data analysis, genomic DNA extraction, and TaqMan copy number assay

Our method for CNV analysis of WGS data was described in our previous report.^22^ To extract genomic DNA from tissues and cells for copy number assay, we used the QIAamp DNA mini kit (Qiagen, German) according to the manufacturer's protocols. We quantified the genomic DNA using a NanoDrop 2000 spectrophotometer (Thermo Fisher Scientific, Waltham, MA, USA). qRT‐PCR was performed using a TaqMan copy number assay kit (ThermoFisher Scientific) according to the manufacturer's protocols. RNase P was co‐amplified and used as an internal control (TaqMan Copy Number Reference Assay). Assays were performed on an ABI PRISM™7900 HT Sequence Detection System (SDS; Applied Biosystems, Waltham, MA, USA). The CopyCaller^®^ Software V2.1 (Applied Biosystems) was used with a manual Ct threshold of 0.2 and automatic baseline to analyze the real‐time quantification data from the SDS software. The predesigned TaqMan probes are shown in Table S5.

### 5.4 Vectors and cell transfection

All vectors were obtained from Shanghai GeneChem Co. (Shanghai, China). HepG2 and PLC/PRF/5 cells were transfected with LINC01133 overexpression (Ubi‐MCS‐SV40‐Cherry‐LINC01133) or control vectors. MHCC97H and HCCLM3 cells were transfected with shRNA against LINC01133 (hU6‐MCS‐CMV‐RFP‐SV40‐Neomycin‐shLINC01133, target sequence: 5′‐TGGCTCTACCTTAAGTCTTTA‐3′) or with control shRNA (target sequence: 5′‐TTCTCCGAACGTGTCACGT‐3′) according to the manufacturer's instructions. The miR‐199a‐5p expression vector (Ubi‐EGFP‐MCS‐IRES‐Puromycin‐miR‐199a‐5p) or corresponding control vector was transfected into MHCC97H and PLC/PRF/5‐LINC01133 cells. The anti‐miR‐199a‐5p (hU6‐MCS‐Ubiquitin‐EGFP‐IRES‐puromycin‐anti‐miR‐199a‐5p, target sequence: 5′‐GAACAGGTAGTCTGAACACTGGG‐3′) or corresponding negative control vector (target sequence: 5′‐TTCTCCGAACGTGTCACGT‐3′) were transfected into PLC/PRF/5 and MHCC97H‐shLINC01133 cells according to the manufacturer's protocols. The ANXA2 expression vector (CMV‐MCS‐3FLAG‐SV40‐Neomycin‐ANXA2) or corresponding control vector was transfected into PLC/PRF/5 cells. The ANXA2 (hU6‐MCS‐Ubiquitin‐EGFP‐IRES‐puromycin‐shANXA2, target sequence: 5′‐CTGTACTA TTATATCCAGCAA‐3′) or corresponding negative control vector (target sequence: 5′‐TTCTCCGAACGTGTCACGT‐3′) was transfected into MHCC97H cells according to the manufacturer's protocols. The stability of all transfections was validated by qRT‐PCR.

### 5.5 MicroRNA sequencing

MiR‐seq was performed as described previously.^5^ Briefly, total RNA was extracted from PLC/PRF/5‐Control, PLC/PRF/5‐LINC01133, MHCC97H‐Control, and MHCC97H‐shLINC01133 cells using TRIzol reagent (Invitrogen, Carlsbad, CA, USA). The TruSeq^®^ miRNA Sample Prep Kit v2 (Illumina) was used to prepare RNA‐seq libraries. The RNA was then sent to Genergy Biotechnology Co., Ltd. (Shanghai) for deep sequencing on an Illumina HiSeq 2500 system (Illumina Inc., San Diego, CA, USA).

### 5.6 RNA isolation, reverse‐transcription PCR, and quantitative real‐time PCR (qRT‐PCR)

First, miRNA was isolated using a miRcute miRNA isolation kit (Tiangen, Beijing, China). First‐strand cDNA was then generated using a miRcute miRNA cDNA synthesis kit (Tiangen) and detected using a miRcute Plus miRNA qPCR kit according to the manufacturer's protocols. Forward primers of miR‐199a‐5p (cat.CD201‐0272), miR‐501‐5p (cat.CD201‐0641), and U6 snRNA (cat.CD201‐0145) were purchased from TIANGEN BIOTECH CO., LTD. The relative miRNA expression was normalized to U6 expression.

Total RNA was isolated from HCC cell lines or tissue samples using Invitrogen TRIzol. A PARIS kit (Life Technologies, Carlsbad, CA, USA) was used to separate and purify cytoplasmic and nuclear RNA. A NanoDrop 2000 spectrophotometer was used to determine the extracted RNA concentration. For reverse‐transcription PCR, cDNA was synthesized using a cDNA reverse transcription kit (Applied Biosystems, Carlsbad, CA, USA). Then, qRT‐PCR was conducted on an ABI PRISM™7900 HT sequence detection system (Applied Biosystems) using SYBR Green I (Takara, Japan). The primers for lincRNAs and other genes are summarized in Table S6. The relative expression of lincRNAs and mRNAs was normalized to 18s rRNA expression and GAPDH expression, respectively. The RNA expression was quantified using the 2^−Δ^ *^Ct^* (Δ*Ct* = *Ct* (Target) – *Ct* (Reference)) method.

### 5.7 RNAscope assay

Subcellular localization of LINC01133 was analyzed by RNAscope assay. HCC cells and tissue samples were fixed in 10% formaldehyde for 24 hours. After rinsing with phosphate buffered saline, the cells or tissue samples were treated with histogel solution, dehydrated, embedded into a paraffin block, and cut into sections 7‐15 μm thick. The RNAscope assay was conducted using an RNAscope^®^ 2.5 HD detection kit (Advanced Cell Diagnostics, CA, USA) with the RNAscope® Probe Hs‐LINC01133 (Cat.456551, Advanced Cell Diagnostics, CA, USA) according to the manufacturer's protocols.^51^ Photographs were taken with high‐power magnification (100×, 200×, 400×) on an Olympus microscope (Olympus, Japan).

### 5.8 Cell‐proliferation, colony‐formation, cell‐migration, and invasion assays

HCC cells were seeded at a density of 1000 cells per well on a 96‐well plate and cultured for 24 hours. Then, 10 μL CCK‐8 solution (Dojindo, Japan) was added to the wells at different time points, and the cells were incubated for 2 hours. The optical density was then measured at 450 nm to determine the cell viability in each well.

For the colony‐formation assays, HCC cells were plated on a six‐well plate (1000 cells/well) and maintained in DMEM supplemented with 10% FBS for 2 weeks. The resulting colonies were then fixed with methanol and stained with 0.1% crystal violet. Photographs were captured, and the colonies were quantified using Image J software.

Cell‐migration and invasion assays were carried out on 24‐well plates with transwell inserts (8‐μm pore size; Millipore, Billerica, MA, USA). Matrigel‐coated (Millipore) inserts were used in the invasion assay. In each assay, 1 × 10^5^ HCC cells were suspended in 350 μL serum‐free DMEM in the upper chamber of the wells, and 700 μL DMEM with 10% FBS was placed in the lower chamber. The cells were incubated for 24‐48 hours, and the migrating or invading cells on the lower membrane surface were fixed in 4% paraformaldehyde and stained with crystal violet. The invading or migrating cells were counted in five randomly selected fields under a microscope (Olympus, Japan). Images were acquired at 200× magnification.

### 5.9 Luciferase reporter assay

Wild‐type or mutant LINC01133 or SNAI1 3′‐UTR was inserted downstream of the luciferase reporter gene in the pmirGLO vector (Promega, Madison, WI, USA). Human HEK‐293T cells were cotransfected with miR‐199a‐5p mimics or control RNA (Invitrogen) along with the constructed reporter vectors using Invitrogen Lipofectamine 2000. After 48 hours, luciferase activity was determined using a Dual‐Lumi™ II luciferase reporter gene assay kit (Promega). The relative luciferase activity was calculated as the ratio of firefly luciferase activity to renilla luciferase activity.

### 5.10 RNA pull‐down assay

Sense and antisense transcripts of LINC01133 were obtained using T7 RNA polymerase, labeled using a biotin labeling kit (BersinBio, Guangzhou, China), and incubated for 2 hours with cell lysates at 25°C. Centrifugal enrichment was conducted using streptavidin‐conjugated agarose beads. SDS‐PAGE was performed to separate the precipitated components, followed by silver staining, band isolation, and mass spectrometry (LTQ Orbitrap XL; Thermo Scientific, USA).

### 5.11 RNA immunoprecipitation

RIP was conducted to investigate the interaction of LINC01133 with ANXA2 in MHCC97H cells using the Life Technologies Dynabeads Protein G Immunoprecipitation kit based on the manufacturer's protocols. First, we lysed the MHCC97H cells and incubated the cell lysis solution with antibody‐conjugated protein G magnetic beads at room temperature for 1 hour, during which the magnetic beads conjugated to the anti‐Annexin A2 antibody (cat.11256‐1‐AP, Proteintech, 1:140) or the control rabbit IgG (cat.2729, CST; 1:500). To remove the proteins, the beads were washed with wash buffer and incubated with 0.1% SDS and 0.5 mg/mL proteinase K for 30 minutes at 55°C. Then, qRT‐PCR was performed to analyze the immunoprecipitated RNA.

### 5.12 Protein extraction and western blot

Western blot was performed as previous study.^6^ In short, total proteins were obtained from HCC cells or tissue samples and dissolved in radio immunoprecipitation assay lysis buffer with phenylmethanesulfonyl fluoride (Beyotime, China) and phosphatase Inhibitor Cocktail 2 (Sigma, USA). The proteins were separated by 10% SDS‐PAGE, transferred to Millipore polyvinylidene difluoride membranes, incubated with appropriate antibodies, and detected using a Millipore enhanced chemiluminescence assay kit. The primary antibodies included: anti‐Annexin A2 (cat.8235, CST; 1:1000), anti‐p‐Annexin A2 (Cat.Sc‐135753, Santa Cruz; 1:500), anti‐snail (cat.3879, CST; 1:1000), anti‐cyclin D1 (cat.2922, CST; 1:1000), anti‐E‐cadherin (cat.3195, CST; 1:1000), anti‐N‐cadherin (cat.13116, CST; 1:1000), anti‐vimentin (cat.5741, CST; 1:1000), anti‐STAT3 (cat.9139, CST; 1:1000), anti‐p‐STAT3(Tyr705) (cat.9145, CST; 1:1000), anti‐GAPDH (cat.5174, CST; 1:1000), and rabbit IgG (cat.2729, CST; 1:1000).

### 5.13 Tissue microarray and immunohistochemistry assay

TMAs were constructed as previously described.^52^ Briefly, two core biopsies 2 mm in diameter were taken from donor blocks and transferred to a recipient paraffin block at defined array positions. The TMA was constructed using the tissue samples from the 380 patients in cohort 3. IHC staining was performed using the avidin‐biotin‐peroxidase complex method. Briefly, after rehydration and microwave antigen retrieval, anti‐Annexin A2 (cat.8235, CST; 1:200) was applied to slides, and the slides were incubated at 4°C overnight. Then, the slides were incubated with secondary antibody (GK500705, Gene Tech, Shanghai, China) at 37°C for 30 minutes. Staining was carried out with 3,3′‐diaminobenzidine (DAB). Mayer's hematoxylin was used for counterstaining. Images of representative fields were captured under a microscope at 200× magnification (Olympus, Japan). The ANXA2 densities were counted using the Image‐Pro Plus v 6.2 software (Media Cybernetics, Inc., Bethesda, MD, USA). The integrated optical density of all positive staining was measured in each photograph. The ratio of the stained area to the total area of each photograph was calculated as the ANXA2 density. The median densities were used as the cutoff values to analyze their association with the clinical outcomes in cohort 3. Patients with an ANXA2 density higher or lower than the median ANXA2 density were classified as ANXA2^high^ or ANXA2^low^, respectively. Negative control slides with the primary antibodies omitted were included in all assays.

### 5.14 In vivo tumor growth and pulmonary metastasis assays

PLC/PRF/5‐Control, PLC/PRF/5‐LIN01133, MHCC97H‐shControl, and MHCC97H‐shLINC01133 cells (1 × 10^7^) were suspended in a 1:1 volumetric mixture of 100 μL serum‐free DMEM (Gibco, USA) and Matrigel (BD Biosciences) and injected subcutaneously into the upper left flank of 4‐week‐old nude mice (two mice/group). After 4 weeks of feeding, the nude mice were sacrificed by cervical vertebrae removal. The tumors (2 × 2 × 2 mm^3^) were then inoculated onto the livers of 6‐week‐old nude mice (six mice/group). After 6 weeks of rearing, the mice were sacrificed by cervical dislocation, and the tumor dimensions were measured using a digital caliper. The tumor volume (mm^3^) was calculated as follows: *V* = *ab* ^2^⁄2, where a and b are the largest and smallest tumor diameters measured at necropsy, respectively.^53^ Then, the lungs were embedded in paraffin, and the total number of lung metastases was counted under a microscope as described previously.^54^ The metastases were classified into four grades on the basis of the number of tumor cells present at the maximal section of each metastatic lesion: grade I, < 20 tumor cells; grade II, 20‐50 tumor cells; grade III, 50‐100 tumor cells; grade IV, > 100 tumor cells. The immunoreactivity of the prepared tumor tissue sections was analyzed as described above using anti‐Ki‐67 (cat.ab16667, Abcam; 1:100), anti‐E‐cadherin (cat.3195, CST; 1:400), anti‐N‐cadherin (cat.13116, CST; 1:200), and anti‐vimentin (cat.5741, CST; 1:400).

### 5.15 Statistical analysis

Statistical analysis was conducted using SPSS 20.0 (Chicago, IL, USA) for Windows or GraphPad Prism V7 (GraphPad, La Jolla, CA, USA). Measured values were expressed as the mean ± standard deviation (SD). Normally distributed data were analyzed using two‐tailed Student's *t*‐test or analysis of variance. Categorical variables were compared by Chi‐square test or Pearson's test. Correlation between two variables was analyzed using Pearson's coefficient. Relationships with clinicopathological characteristics were analyzed by chi‐square test or Fisher's exact test. The Kaplan‐Meier method followed by log‐rank test was used for survival analysis. We considered *P* values < 0.05 to be statistically significant.

## AUTHOR CONTRIBUTIONS

DY, ZQH, CBL, and XYW contributed equally to the study. DY, ZQH, CBL, XYW, and JL performed the experiments. DY, ZQH, and SLZ analyzed and interpreted the data. HYX, RQS, and PCW provided the tissue samples and the clinical data. DY, SLZ, and ZJZ drafted the manuscript. JF and JZ commented on the study and revised the paper. ZJZ, JZ, and SLZ obtained funding and designed the research. All authors read and approved the final manuscript.

## AVAILABILITY OF DATA AND MATERIALS

All data in our study are available upon request.

## ETHICS APPROVAL AND CONSENT TO PARTICIPATE

The Research Ethics Committee of Zhongshan Hospital granted ethical approval for the use of human subjects. All participants gave informed consent to be included in the study.

Animal studies in nude mice were approved by the Institutional Animal Experiment Committee of Zhongshan hospital and carried out in accordance with the “Guide for the Care and Use of Laboratory Animals” prepared by the National Academy of Sciences and published by the National Institutes of Health (NIH publication 86‐23 revised 1985).

## CONFLICT OF INTEREST

The authors declare that they have no competing interests.

## Supporting information

FIGURE S1

FIGURE S2

figurelegends

TABLES S1‐S6
