# Supplementary figures and images for: LINC01133 promotes hepatocellular carcinoma progression by sponging miR‐199a‐5p and activating annexin A2
Source: Clin Transl Med. 2021 May 6;11(5):e409. doi: 10.1002/ctm2.409 (PMC8101537; doi:10.1002/ctm2.409)

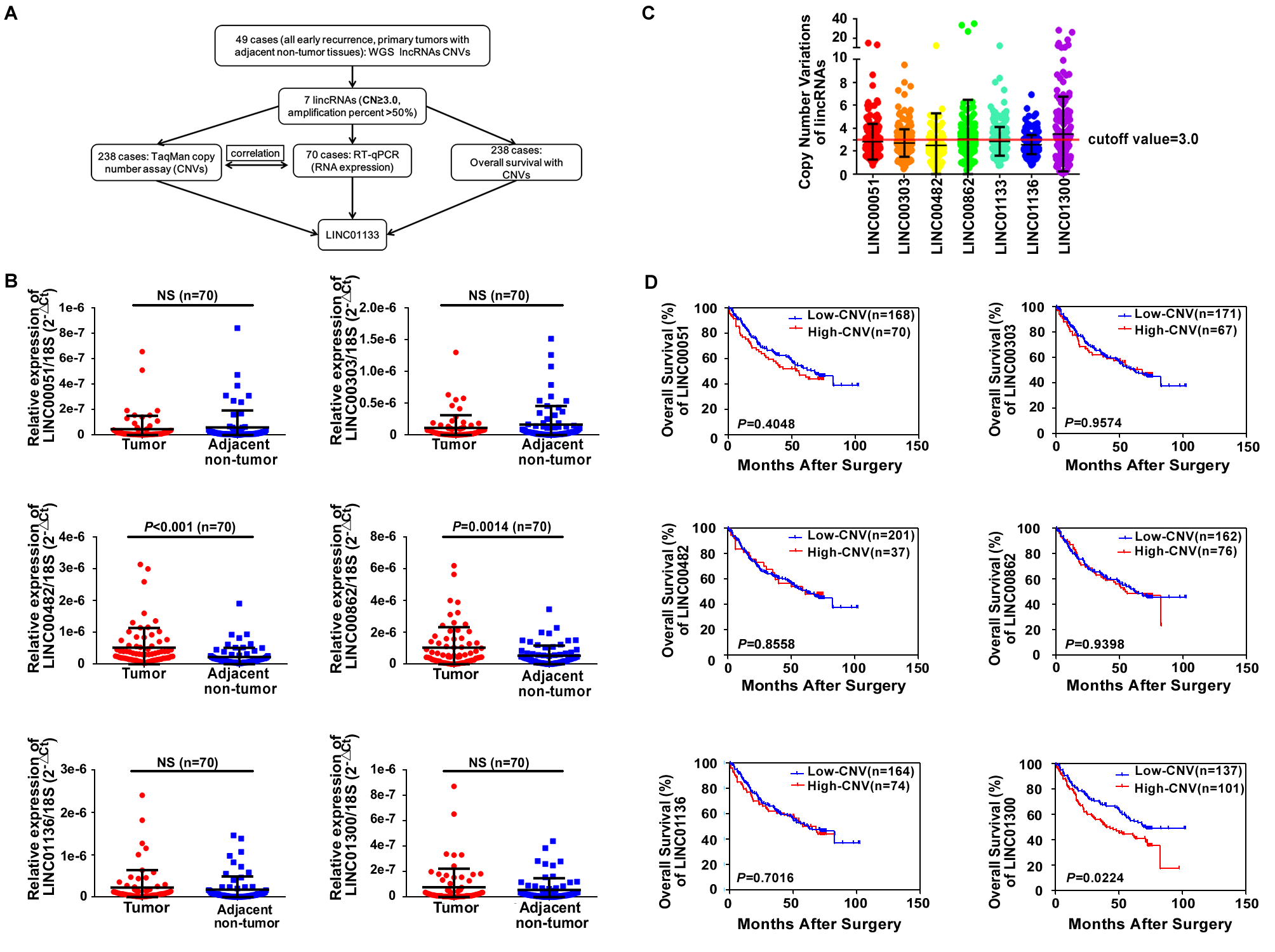

Supplement: Supplementary file 1 — FIGURE S1 [file CTM2-11-e409-s002.tif]

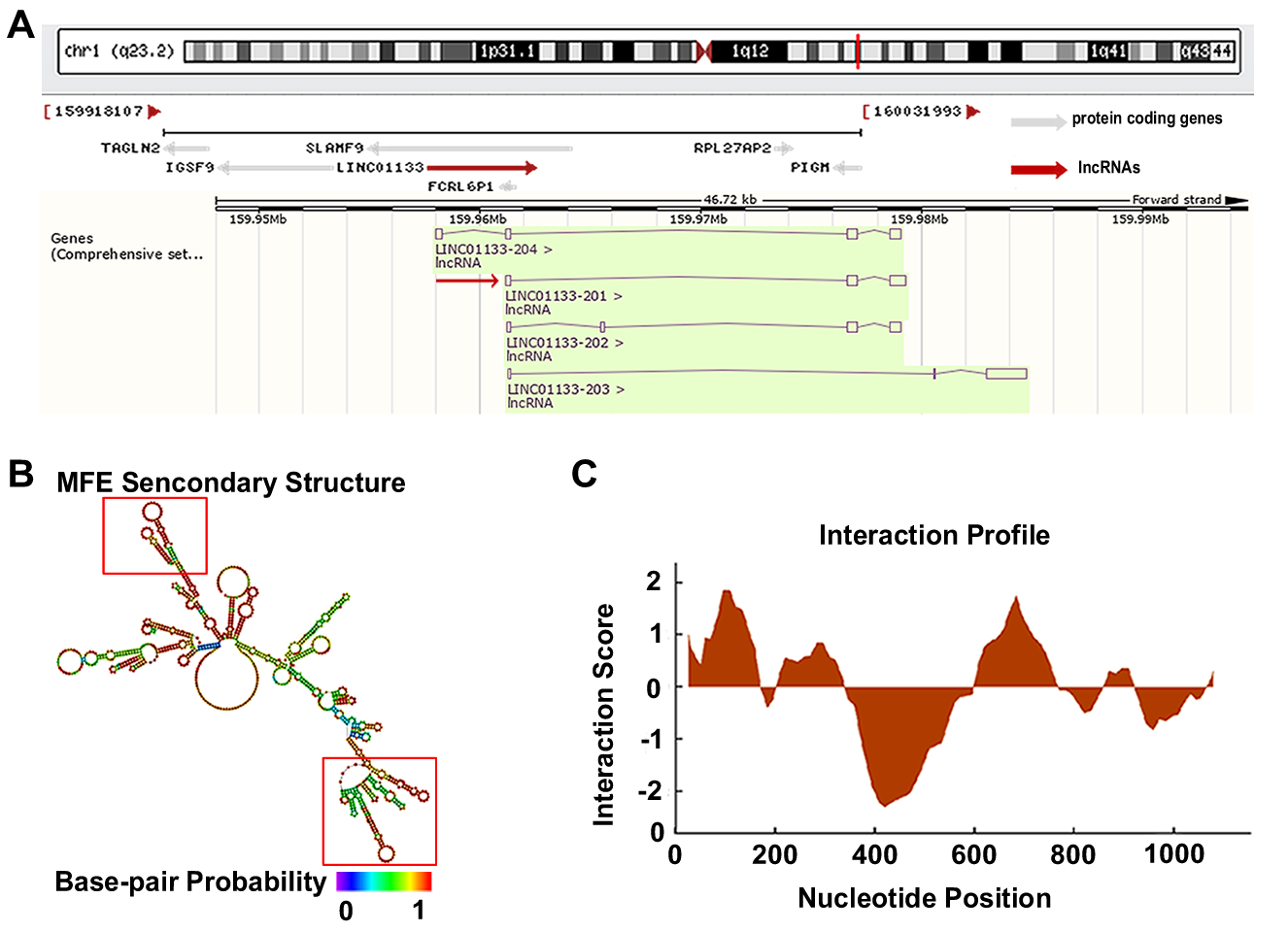

Supplement: Supplementary file 2 — FIGURE S2 [file CTM2-11-e409-s003.tif]
