# Supplementary material for: LINC01133 promotes hepatocellular carcinoma progression by sponging miR‐199a‐5p and activating annexin A2
Source: Clin Transl Med. 2021 May 6;11(5):e409. doi: 10.1002/ctm2.409 (PMC8101537; doi:10.1002/ctm2.409)
Supplement: Supplementary file 3 — figurelegends [file CTM2-11-e409-s004.docx]

**Supplementary Figure legends**

**Supplementary Figure 1. Identification of the LINC01133 with CNVs involved in HCC recurrence and prognosis. (A)** The flow chart for selecting candidate lincRNAs in HCC is shown. LINC01133 was identified for further study. **(B)** qRT-PCR was performed to determine the expression levels of LINC00051, LINC00303, LINC00482, LINC00862, LINC01136 and LINC01300 in 70 pairs of HCC samples and adjacent non-tumor tissue samples. 18S rRNA was used as an internal reference. **(C)** The CNV of seven candidate lincRNAs in 238 paired tissue samples was determined by TaqMan copy number assay. RNase P was used as an internal reference. The red line indicates the cutoff value (the cutoff value of CNVs=3.0). **(D)** Kaplan-Meier analyses of overall survival based on CNVs in LINC00051, LINC00303, LINC00482, LINC00862, LINC01136 and LINC01300 in 238 patients (cohort 2). Data are shown as the mean ± standard deviation.

**Supplementary Figure 2. The role of LINC01133 in HCC. (A)** The genomic localization (<https://www.ncbi.nlm.nih.gov/gene/>) and transcripts of LINC01133 (<http://asia.ensembl.org/>). **(B)**The potential ANXA2-binding site on LINC01133 in the secondary structure diagram was predicted by RNAfold web server (<http://rna.tbi.univie.ac.at/cgi-bin/RNAWebSuite/RNAfold.cgi>). **(C)** The potential ANXA2-binding site on LINC01133 was predicted using catRAPI omics (<http://service.tartaglialab.com/page/catrapid_omics_group>).
