# Supplementary material for: LINC01133 promotes hepatocellular carcinoma progression by sponging miR‐199a‐5p and activating annexin A2
Source: Clin Transl Med. 2021 May 6;11(5):e409. doi: 10.1002/ctm2.409 (PMC8101537; doi:10.1002/ctm2.409)
Supplement: Supplementary file 4 — TABLES S1‐S6 [file CTM2-11-e409-s001.docx]

**Supplementary Tables**

**Table S1:** **The detailed information of seven candidate lincRNAs**

| Name | LincRNAs ID | Chr. | CNVs Percent  (% in 49 cases) | Average  copy number |
| --- | --- | --- | --- | --- |
| LINC01133 | ENSG00000224259 | 1 | 61.22 (30/49) | 3.96 |
| LINC00862 | ENSG00000203721 | 1 | 69.39 (34/49) | 3.71 |
| LINC01136 | ENSG00000233791 | 1 | 69.39 (34/49) | 3.77 |
| LINC00303 | ENSG00000176754 | 1 | 69.39 (34/49) | 3.71 |
| LINC01300 | ENSG00000253595 | 8 | 63.27 (31/49) | 4.10 |
| LINC00051 | ENSG00000254008 | 8 | 63.27 (31/49) | 4.25 |
| LINC00482 | ENSG00000185168 | 17 | 51.02 (25/49) | 3.35 |

**Table S2: Correlation between the factors and clinicopathologic characteristics in HCC (Cohort 2, n =238)**

| Clinicopathological Indexes | | CNV in LINC01133 | | *P* value ^†^ |
| --- | --- | --- | --- | --- |
|  |  | Low(n=159) | High(n=79) |  |
| Age(year) | ≤ 50 | 71 | 38 | 0.615 |
|  | >50 | 88 | 41 |  |
| Sex | Female | 23 | 9 | 0.513 |
|  | Male | 136 | 70 |  |
| HBsAg | Negative | 22 | 11 | 0.985 |
|  | Positive | 137 | 68 |  |
| AFP (ng/ml) | ≤20 | 56 | 28 | 0.973 |
|  | >20 | 103 | 51 |  |
| GGT(U/L) | ≤54 | 67 | 30 | 0.538 |
|  | >54 | 92 | 49 |  |
| Liver cirrhosis | No | 36 | 18 | 0.980 |
|  | yes | 123 | 61 |  |
| Tumor size(cm) | ≤5 | 80 | 34 | 0.290 |
|  | >5 | 79 | 45 |  |
| Tumor number | Single | 135 | 65 | 0.602 |
|  | Multiple | 24 | 14 |  |
| Microvascular invasion | Absence | 148 | 66 | **0.021** |
|  | Present | 11 | 13 |  |
| Tumor encapsulation | None | 64 | 31 | 0.881 |
|  | Complete | 95 | 48 |  |
| Tumor differentiation ^‡^ | I+II | 109 | 45 | 0.078 |
|  | III+IV | 50 | 34 |  |

† Chi-square tests for all analyses. ‡ Edmondson grade. *P* values less than 0.05 were considered statistically significant. Abbreviations: CNV, copy number variation; HBsAg, hepatitis B surface antigen; AFP, alpha-fetoprotein; GGT, gamma-glutamyl transpeptidase.

**Table S3: Prediction of LINC01133 protein-coding ability (LNCipedia Database,** <https://lncipedia.org/>**)**

| Metric | Raw Result | Interpretation |
| --- | --- | --- |
| PRIDE reprocessing 2.0 | 0 | non-coding |
| Lee translation initiation sites | 0 | non-coding |
| CPAT coding probability | 2.09% | non-coding |
| PhyloCSF score | -167.9717 | non-coding |
| Bazzini small ORFs | 0 | non-coding |

**Table S4: Clinicopathologic characteristics of HCC patients**

| Clinicopathologic  characteristics | | Cohort 1  (n=49) | | Cohort 2  (n=238) | | Cohort 3  (n=380) | |
| --- | --- | --- | --- | --- | --- | --- | --- |
|  |  | n | % | n | % | n | % |
| Age(year) | ≤ 50 | 12 | 24.5 | 109 | 45.8 | 150 | 39.5 |
|  | >50 | 37 | 75.5 | 129 | 54.2 | 230 | 60.5 |
| Sex | Female | 12 | 24.5 | 32 | 13.4 | 65 | 17.1 |
|  | Male | 37 | 75.5 | 206 | 86.6 | 315 | 82.9 |
| HBsAg | Negative | 7 | 14.3 | 33 | 13.9 | 54 | 14.2 |
|  | Positive | 42 | 85.7 | 205 | 86.1 | 326 | 85.8 |
| AFP (ng/ml) | ≤ 20 | 16 | 32.7 | 84 | 35.3 | 145 | 38.2 |
|  | >20 | 33 | 67.3 | 154 | 64.7 | 235 | 61.8 |
| GGT(U/L) | ≤54 | 16 | 32.7 | 97 | 40.8 | 195 | 51.3 |
|  | >54 | 33 | 67.3 | 141 | 59.2 | 185 | 48.7 |
| Liver cirrhosis | Yes | 41 | 83.74 | 54 | 22.7 | 61 | 16.1 |
|  | No | 8 | 16.3 | 184 | 77.3 | 319 | 83.9 |
| Tumor size(cm) | ≤ 5 | 31 | 63.3 | 114 | 47.9 | 232 | 61.1 |
|  | >5 | 18 | 36.7 | 124 | 52.1 | 148 | 38.9 |
| Tumor number | Single | 45 | 91.8 | 200 | 84.0 | 337 | 88.7 |
|  | Multiple | 4 | 8.2 | 38 | 16.0 | 43 | 11.3 |
| Microvascular invasion | Absence | 28 | 57.1 | 214 | 89.9 | 261 | 68.7 |
|  | Present | 21 | 42.9 | 24 | 10.1 | 119 | 31.3 |
| Tumor encapsulation | Complete | 29 | 59.2 | 95 | 39.9 | 211 | 55.5 |
|  | None | 20 | 40.8 | 143 | 60.1 | 169 | 44.5 |
| Tumor differentiation | I+II | 37 | 75.5 | 154 | 64.7 | 285 | 75.0 |
|  | III+IV | 12 | 24.5 | 84 | 35.3 | 95 | 25.0 |

Abbreviations: HBsAg, hepatitis B surface antigen; AFP, alpha-fetoprotein; GGT, gamma-glutamyl transpeptidase.

**Table S5：Probes used in this study (Applied Biosystems, USA)**

| Name | Assay ID | Cytoband | Amplicon Length |
| --- | --- | --- | --- |
| LINC00051 | Hs06227552_cn | 8q24.3 | 105bp |
| LINC00303 | Hs01097482_cn | 1q32.1 | 108bp |
| LINC00482 | Hs01830205_cn | 17q25.3 | 98bp |
| LINC00862 | Hs05714331_cn | 1q32.1 | 102bp |
| LINC01133 | Hs04523544_cn | 1q23.2 | 104bp |
| LINC01136 | Hs05783302_cn | 1q32.1 | 98bp |
| LINC01300 | Hs03665520_cn | 8q24.3 | 100bp |
| RNase P | Reference Assay | 14q11.2 | 88bp |

Table S6：Primer sequences used in this study

| Name | Forward primer (5’ →3’) | Reverse primer (5’→3’) |
| --- | --- | --- |
| 18S | CGGACAGGATTGACAGATTGATAGC | TGCCAGAGTCTCGTTCGTTATCG |
| LINC00051 | CTCTTCTCCCAAGAGCGAGTG | AGGTCAGGTAACATTGCTGGG |
| LINC00303 | GTACCGTTGTCCTGGGAGTG | GGCTGAGTGCCAAACAAGTG |
| LINC00482 | GGCTCTGCCGACAGCTATTT | CAAGAACAAGGGGCTGGGTT |
| LINC00862 | TGTGGCTGATGTGCATTTTGG | GCTTTGTAGCCATCCGGTGT |
| LINC01133 | GCTGTGGTGGAGAGAATGGA | CCCCAGCTTTCCAGATCCAAA |
| LINC01136 | AGTTCTAGGCCAGCCAGTCT | GCATCCCCACCTTCCTGTAG |
| LINC01300 | TGGTGAATGTGGCTTCTCTG | CCTGCACTGGGCATTATCTT |
| GAPDH | AAGGTGAAGGTCGGAGTCAAC | GGGGTCATTGATGGCAACAATA |
| ANXA2 | CTCTACACCCCCAAGTGCAT | TCAGTGCTGATGCAAGTTCC |
| E-cadherin | AGCCCCGCCTTATGATTCTCTG | TGCCCCATTCGTTCAAGTAGTCAT |
| N-cadherin | ATTTGAGGGCACATGCAGTAG | GAACTGTCCCATTCCAAACCT |
| vimentin | CGCCAGGCAAAGCAGGAGTC | TGCAGGCGGCCAATAGTGTCT |
| snail | TCGGAAGCCTAACTACAGCGA | AGATGAGCATTGGCAGCGAG |
| U1 | GAAACTCGACTGCATAATTTGTGGTAG | CTTGGCGTACAGTCTGTTTTTGAAACTC |
